# Rental Market Risk and Radical Right Support

**DOI:** 10.1177/00104140241306963

**Published:** 2024-12-08

**Authors:** Tarik Abou-Chadi, Denis Cohen, Thomas Kurer

**Affiliations:** 1 6396 University of Oxford, Oxford, UK; 2 26573 University of Mannheim, Mannheim, Germany; 3 30965 University of Zurich, Zurich, Switzerland

**Keywords:** housing, rental markets, far right, elections, social status, economic risk

## Abstract

A growing literature examines how economic threat affects support for anti-establishment parties. While most existing work focuses on transforming labor markets as a source of anxiety, we advance the literature by studying changes in urban development and rent price appreciation. Our analysis examines the case of Germany, the country with the highest share of rental housing in the European Union. Combining individual-level geo-referenced panel data with a longitudinal data set on the cost of rental housing at the postcode level, we demonstrate that rising local rent levels increase support for radical right parties among long-term residents with lower household incomes, in particular in urban areas. Our results suggest that urban development, not unlike labor market transformation, represents an important and so far neglected source of economic insecurity and social concern with important political implications.

## Introduction

The share of people who live in rented accommodation is on the rise in most post-industrial democracies. Almost everywhere, increases in rent levels and housing affordability are a key source of economic pressure. According to Eurostat Housing Statistics, the proportion of the EU population that suffers from housing cost overburden (exceeding 40% of their equivalized disposable income) is by far highest for tenants with market price rents.

It is thus unsurprising that rent price developments are a major concern among citizens in many European democracies and beyond. In a representative long-term study on political, economic, environmental and personal fears in Germany, affordable housing consistently features among the top-3 issues. In 2023, West Germans in particular are concerned about the tight situation on the housing market (62%), while the figure for East Germans is 52%.^1^

Despite their real-world importance, social science research has only recently begun to uncover the consequences of the affordability crisis in the rental market (Fetzer et al., 2023; Held & Patana, 2023; Hilbig & Wiedemann, 2023). The political implications of housing have so far been primarily studied from the perspective of homeowners (Adler & Ansell, 2020; Ansell, 2014, 2019; Larsen et al., 2019). However, we argue here that the political implications of rental markets differ fundamentally from what we know about homeownership. In this article, we build on the existing literature on status threat (Gidron & Hall, 2017; Kurer, 2020) and introduce the concept of *rental market risk* to better understand the relationship between rental housing and electoral behavior. We demonstrate that rental market risk systematically affects individual party preferences of tenants in ways that contrast starkly with the behavior of homeowners. Rental market risk describes individuals’ exposure to the latent economic threat from a rent price appreciation in their local environment. Importantly, local rental market dynamics are entirely beyond the control of individual tenants. Even when local rent price appreciation has not (yet) affected individuals’ own rents, it signals that the local environment is in flux, a development that may go hand in hand with an imminent increase in one’s cost of living – an increase that not all can afford. Local rent price appreciation hence represents a profound and previously neglected source of economic risk, which we expect to affect electoral preferences.

In developing our argument, we emphasize the role of households’ economic resources and residential geography. Building on an emerging literature on the political ramifications of economic risk and status anxiety (Abou-Chadi & Kurer, 2021; Engler & Weisstanner, 2021; Gidron & Hall, 2017; Kurer, 2020), we anticipate that rental market risk, conceptualized as a latent threat from rising local rent levels, fuels support for the radical right. This party family has been shown to successfully appeal to voters who are concerned about their potential *relative* decline in the social hierarchy even though other parties, notably the Left, may actually look like a more obvious political choice when considering specific programmatic priorities in the domain of housing market regulation.

We expect that tenants in the lower half of the income distribution will be particularly likely to support the radical right when local rents go up. They face higher rental market risk because lower income deciles typically spend a higher share of their disposable income on housing (Fetzer et al., 2023). We argue that household income is a crucial factor that separates those for whom rising rent prices pose severe economic and status threats from beneficiaries of neighborhood upgrading. Higher income increases the chances that individuals will be able to afford imminent increases in their household rents. It thereby shields tenants from the threat of being driven out of their neighborhood. The lower people’s income, on the other hand, the more they will perceive increases in rental prices as a threat. Furthermore, we argue that this mechanism will be particularly pronounced where rent spikes have been most frequent and most forceful: In booming regions, in cities and metropolitan suburbia, where strong rent increases often materialize rapidly and are more vividly perceived by voters. The rapid change and immediate exposure to increasing rent loads that many face in these areas amplifies the political significance of economic and status threats.

In analyzing the so far neglected aspect of rental market change, our argument thus not only presents a novel perspective on the political effects of the housing market. It also offers a new perspective on place-based drivers of radical right support. Existing research on the structural differences between booming and declining regions shows that residents of rural and stagnant regions disproportionately support the radical right (e.g., Patana, 2022; Rodríguez-Pose, 2018), a pattern that is mostly attributed to compositional socio-structural differences that result from residential self-selection (Maxwell, 2019, 2020). Moving beyond these analyses of inter-regional discrepancies, we zoom in on dynamic changes within places to offer an explanation why radical right parties attract significant shares of the vote even in booming urban areas. By showing that those at risk of being economically overburdened and driven out by neighborhood upvaluation increasingly turn to the radical right, we identify a largely overlooked reservoir of radical right support.

To test our expectations, we rely on an innovative combination of high-quality geo-referenced panel data from the German Socio-Economic Panel (GSOEP) with novel longitudinal and spatially disaggregated information on the cost and quality of rental housing. This allows us to analyze panel data from 2005 to 2018 and to investigate how individuals react to changes in rent levels in their neighborhood. The case of Germany is not only an ideal testing ground for our theory because it has the highest share of renters in the European Union but also due to the political geography of support for the radical right party *Alternative für Deutschland* (AfD). Support for the AfD is strongest in the rural periphery, where it sometimes exceeds 30% of the vote. Accordingly, we can show with our data that – in cross-sectional perspective – support for the party is strongest where rents have been and remained low. Yet, we know that the party also receives significant vote shares in urban and suburban districts. Turning to dynamic changes within neighborhoods, we show how the rental market forcefully drives the far-right vote outside of rural areas.

Rental market dynamics thus constitute an important element for place-based explanations of radical right support. This literature has largely focused on declining regions (e.g., Bolet, 2021), losers of globalization (e.g., Walter, 2021), and general feelings of rural resentment (e.g., Cramer, 2016; Hochschild, 2016). It has thereby often turned a blind eye on radical right success in urban centers and other regions that are not “left behind”. Rental market risk can help us understand why in booming regions, too, we find support for those parties. Beyond its general importance for questions of political behavior and political economy, our argument thus taps into a group so far largely understudied in radical right support: People with limited economic resources who live in comparatively well-off neighborhoods but fear that they may not be able to keep track with the economic dynamism of their place of residence. Given the centrality of rent prices in many people’s lives and the dramatic changes that rental markets have undergone in the past two decades, close examination of this link is crucial for understanding the transformation of political behavior in Europe.

## The Politics of Housing

### Homeownership

The existing body of work on the politics of housing has almost exclusively focused on homeownership. In contrast to the literature on labor market risk with its focus on fluctuations in current income streams (e.g., Iversen & Soskice, 2001; Rehm, 2009; Walter, 2017), the housing literature emphasizes the importance of long-term consumption patterns, which are smoothed over time by assets and wealth and, hence, are at least partly independent from labor market dynamics (Friedman, 1957). Building on this concept of “permanent income”, Ansell (2014) offers a theory of the political preferences of homeowners. Assets provide a stock of wealth that serve as a form of self-insurance against hard times. Accordingly, the ability to “self-insure” against short-term fluctuations in labor market income results in higher tax aversion and lower demand for social insurance and redistribution provided by the government (Ansell, 2014). With respect to party vote choice, this pattern of social policy preferences among homeowners translates into increased support for center-right parties (Ansell & Cansunar, 2021).

Beyond the individual-level pocketbook logic, rising house prices are also a proxy for the relative wealth of a locality and an improving economy more generally (Ansell, 2019). In line with this geotropic perspective, rising house prices at the local or regional level are associated with support for the political status quo. This support manifests itself in a variety of ways, including higher vote shares for the incumbent party (Larsen et al., 2019), support for mainstream parties, in particular from the center-right (Ansell & Cansunar, 2021), and lower support for populist actors challenging the establishment as in the Brexit referendum campaign (Adler & Ansell, 2020).

The geotropic component of the politics of housing also highlights a relevant flipside of the house price appreciation argument with political implications transcending traditional left-right or “first-dimension” politics. Citizens in places where housing prices stagnate or even decline may feel excluded from the massive gains of homeowners in booming localities, and possibly interpret this as a signal that the market does not value places like theirs. Voters in such “left-behind” regions with less dynamic housing markets hence might want to attack the political status quo by supporting anti-establishment and/or populist parties (Adler & Ansell, 2020; Ansell et al., 2022).

For homeowners, the pocketbook effect that operates via individual economic well-being and the geotropic effect that unfolds via the relative wealth of a locality go hand in hand and produce consistent expectations about the political repercussions of house price appreciation.

### Rental Markets

While rising house prices in most European countries directly benefit many homeowners, tenants in the rental market face the other side of that coin. In contrast to the more cyclical housing price market, rent levels have increased steadily throughout the years. According to Eurostat, rents have gone up by 14.6% across the EU-27 during the last ten years. This average masks large variation both between and within countries, with urban areas, which host two-thirds of the European population, seeing particularly pronounced surges.

Despite the importance of rising rent levels as a key source of economic insecurity, researchers have only very recently begun to uncover the political causes and consequences of this development. Most closely related to our work is Held and Patana’s (2023) important recent study on the relationship between personal rent loads and voting behavior. They provide evidence that *actual* household rents fuel support for radical right parties in Germany and argue that an explicitly cultural frame of rising rents, which emphasizes housing market competition with immigrants and refugees, allows radical right parties to mobilize voters on this inherently economic issue. In another relevant contribution, Fetzer et al. (2023) examine the consequences of a cut in housing benefits and show that this shock in the affordability of rents affected a variety of socio-economic outcomes including evictions, crime and homelessness. In addition, they document that exposure to benefit cuts is also associated with lower political participation. Hilbig and Wiedemann (2023) examine the political constraints to the construction of public housing, a central policy remedy to the affordability crisis. Finally, Beckmann et al. (2020) study ‘housing cleavages’ in Germany by examining differences in political behavior between homeowners and tenants and high-price versus low-price areas.

## Rental Market Risk and Electoral Behavior

In developing our theoretical argument, we differentiate between two conceptually distinct mechanisms linking rent levels to political behavior: A pocketbook channel focusing on individual economic circumstances and a geotropic channel focusing on risk perceptions in a changing environment.

In the context of the rental market, the pocketbook channel stipulates that increases in *actual* household rents prompt political reactions among affected individuals. There is some disagreement about the exact mechanism and expected directionality of this effect. From a purely economic, first-dimension politics perspective, the pocketbook mechanisms yields similarly straightforward expectations as in the case of homeownership – just with an opposite sign. All things equal, an increase in *actual* rents at the individual level implies higher expenditures and lower disposable income. A standard political economy perspective would thus expect that voters with lower disposable income support redistribution and political parties of the left. However, adopting a second-dimension politics perspective, one may argue that radical right parties employ a cultural frame, which emphasizes competition with immigrants – in both the private and public rental market – to mobilize affected voters (Cavaillé & Ferwerda, 2023; Held & Patana, 2023).

We argue, however, that the relationship between changing rents and electoral behavior is not limited to the egotropic channel. The present study complements the existing pocketbook perspective with a specific focus on latent *rental market risk*, conceptualized as local rent price appreciation (net of individual household rents). Very much in line with the influential recent literature on the role of status anxiety (Gidron & Hall, 2017; Kurer, 2020), we argue that the rental market affordability crisis has important consequences for the transformation of the political space in post-industrial societies. We propose that increasing rent levels at a granular local level represent an important source of insecurity about voters’ individual capacity to uphold their standard of living and social status in the mid- and long-term. The geotropic effects of changing local rental markets hence entail more than just the flipside of the effects on homeowners discussed above.

First, we need to take into account that rent levels are generally higher in metropolitan areas and university towns. Rent prices are thus highest in those regions that can be seen as the winners of socio-economic change in the knowledge economy. These areas attract skilled workers and experience population growth, which results in housing shortage and rising rents. In that regard, our contribution is markedly different from other important research studying the impact of local economic pressure on far-right voting, for example as a result of Chinese import shocks (Colantone & Stanig, 2018) or long-term decline in manufacturing (Broz et al., 2021). This body of work does a very good job in explaining far-right support in declining regions, typically characterized by an ever smaller number of competitive industries and high levels of out-migration, especially among young people. Importantly, however, these are regions with the lowest levels of rental market pressure because population decline limits the relevance of housing shortages.

The socio-economic make up of booming regions, in contrast, comprises especially those groups with higher levels of education and socio-cultural and managerial occupations (Iversen & Soskice, 2019; Oesch, 2013; Schöll & Kurer, 2024). Given these socio-structural characteristics, local rental prices are likely to correlate with patterns of electoral behavior at the aggregate level, where support for the radical right is expected to be higher in regions with lower rental prices. In contrast, in growing, prosperous regions typically characterized by higher rental prices, we should expect voters disproportionate support for liberal and left-libertarian parties. In this regard, the observable implications of rent levels at the aggregate level are equivalent to those of housing prices.

**Figure 1.**
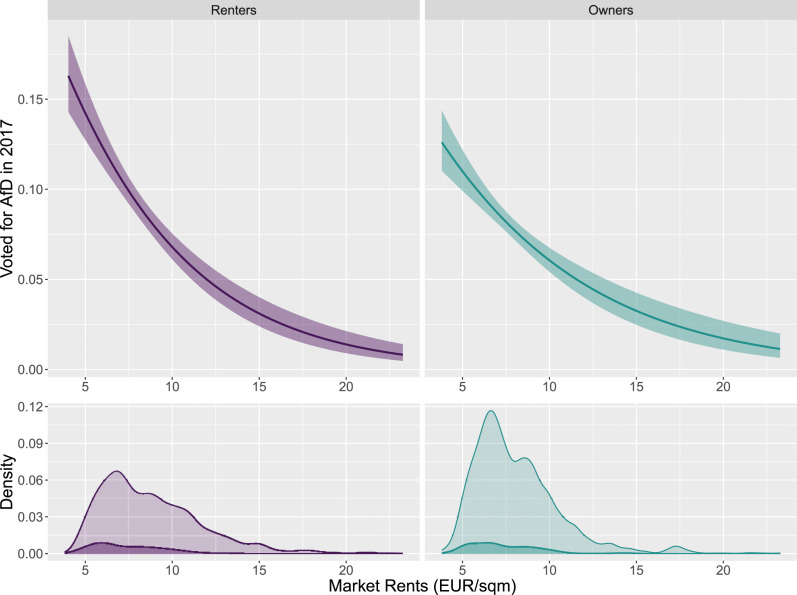
Postcode-level market rents and AfD support in the 2017 German Federal Election. Upper panel: Predicted probability of voting AfD as a function of market rents for renters (left) and homeowners (right) who turned out in the election. Lower panel: Density plots of AfD voters (dark) and non-AfD voters (light).

We can corroborate this cross-sectional point of departure with our own data. Figure 1 shows that support levels for the radical right party AfD are much higher where rents are low compared to places where rents are high. This is in line with the established finding that the radical right is particularly successful in economically struggling regions (Bolet, 2021; Colantone & Stanig, 2018; Rodríguez-Pose, 2018). In Germany in particular, this is also in line with the AfD overperforming in states of the former GDR. Furthermore, Figure 1 also shows that in such a cross-sectional perspective the pattern between homeowners and renters does not differ: AfD support in both groups decreases with rising rents and vice versa.

Hence, looking at the empirical pattern *across* neighborhoods and regions, we find similar results as the existing literature on homeownership (Ansell et al., 2022) and local economic decline (Colantone & Stanig, 2018). Support for the radical right is strongest in areas with low levels of rents. These low-rent areas – located far from the thriving hubs of the knowledge economy and typically characterized by low and declining population density – constitute central strongholds of the radical right. However, this cross-sectional evidence tells us little about the actual effect of rental market pressure. Instead, it tells us something about the effect of well-known socio-demographic and socio-economic predictors of radical right support, whose compositional differences co-determine rent levels and radical right voting in cross-sectional perspective.

To get closer to the causal effect of rental market pressure on party preferences, we need to examine individuals who have lived long enough at a given place to experience the effects of an intensifying rental housing shortage. In such a scenario, we should expect a fundamentally different dynamic. We build on a growing literature that links economic risks and social status to electoral behavior and argue that the relationship between changes in rent levels and political behavior can be best understood in terms of *rental market risk*. The price dynamics of local rental markets are determined by structural forces that are by and large beyond the control of individual tenants. Rising local rent levels represent a profound risk to the many fundamental and sentimentally relevant aspects of a tenant’s existence. The resulting grievances are not only limited to disposable income and the concern to be able to afford the rent. Increasing risks of rental market unaffordability also increase the risk of involuntary relocation, and thereby threaten to disrupt individuals’ established routines and networks: Meeting friends in the local area, visiting one’s favorite shops and restaurants, or enrolling children in local schools. Such uncontrollable local rental market dynamics are likely to prompt individual reactions even in the absence of actual increases in household rents or actual involuntary moves: The latent and intensifying threat of no longer being able to afford living in one’s familiar neighborhood alone may create political grievances. This threat does not only materialize through potential rent increases but can also become relevant in case tenants have to leave their current home due to changing personal circumstances or because of contract terminations.

From this perspective, increases in local rental market prices constitute an economic and social status threat. Building up on existing approaches to status politics in terms of education, occupation or gender, we argue that local rental markets profoundly affect people’s status perceptions. A number of recent contributions show that status threat is an important motive behind the rising support for anti-establishment forces in general, and radical right parties in particular. Support for such parties may not be primarily issue-based. Instead, it stems from a deep and often diffuse discontent, which may have been growing over time. This discontent is rooted in a negative view of the evolution of society and a distinct sense among some citizens that they are increasingly pushed towards the fringes of their community (Bolet, 2021; Elchardus & Spruyt, 2016; Gidron & Hall, 2017; Mutz, 2018; Steenvoorden & Harteveld, 2018). Importantly, this sense of alienation and marginalization is not tantamount to material deprivation but based on perceptions of risk and threat (Kurer, 2020).^2^

We thus expect that where increasing levels of local rents constitute an economic and social status threat, they will translate into support for the radical right. We argue that two factors are crucial for understanding how increasing local rents translate into a status threat and, thereby, increase individuals’ propensity to support the radical right. First, individuals who have been long-term residents in an area should be much more affected by rental market risks. They have built profound ties in their local neighborhoods such that increases in local rents present a much greater threat to their established routines. In addition, people who have recently moved to a neighborhood may have already taken rental market dynamics into account when making their decision to move to an area. Hence, we should expect that increases in local rents primarily lead to support for the radical right among longtime residents.

The second and crucial factor that will determine how increases in local rents affect the likelihood of voting for the radical right is household income. For people with low income, exposure to rising local housing costs represents a largely uncontrollable source of insecurity that threatens to disrupt a seemingly established everyday life. This is true even if one’s own household rent has not (yet) been affected by price hikes. Hence, rising local rents will constitute a status threat especially for people with lower household income. On the contrary, increasing local rents will seem much less of a risk to those with higher incomes. In fact, as long as they can afford it, people with higher income may perceive rent price appreciation and the resulting processes of neighborhood upgrading favorably. Higher rent prices will change the social, economic, and demographic makeup of a neighborhood. For people who can afford it, gentrification, thus, comes with many advantages. These advantages lead to a positive geotropic perception of the neighborhood which in turn should reduce support for populist parties who threaten to disrupt the status quo (Adler & Ansell, 2020; Ansell et al., 2022).

In sum, increasing local rents and the socio-cultural changes of a neighborhood that come along with them will affect low and high-income households fundamentally differently. For low-income households, these changes constitute a profound threat to their social status and should thus make them more likely to support the radical right. For high-income households, the feeling of neighborhood upgrading and the resulting status boost should make them less likely to support the radical right. Our main hypothesis thus states:

H1 *Increases in local market rents increase the probability of supporting the radical right among low-income renters.*

We expect that these diverging effects are particularly strong in urban areas, where phenomena associated with gentrification are particularly pronounced. In urban areas, increases in rent levels have not only been the largest but change has often happened very rapidly. Therefore, changes in status are more easily observable in day-to-day life. Hence, in contrast to a literature that has primarily focused on vastly static, structural characteristics of left-behind regions, we describe a dynamic mechanism that connects booming regions to support for the radical right. People with low income in dynamic urban rental markets face a profound risk of seeing the basic pillars of their life disrupted. As economic insecurity is a well-documented source of alienation and anti-elite sentiment, we expect a strong increase in the propensity to support the radical right in this group.

H2 *The positive effect of local markets rents on the probability of supporting the radical right among low-income renters is strongest in urban areas.*

An important additional observable implication of our argument is that homeowners, in contrast to renters, should be largely shielded from the adverse implications of structural urban development and rent price appreciation. Even if homeowners do not approve of the changing composition of their neighborhood, a potential move out of a booming neighborhood remains entirely their own decision, and selling or leasing their property will most likely be financially beneficial for them. As house price and rent level appreciation benefits homeowners, we should expect it to result in the status-quo enhancing political choices discussed in the section on political implications of homeownership. As a corollary, if we found that changing local rental prices did not affect the electoral behavior of homeowners (or at least not in ways similar to renters), this would strengthen our argument that rental market risks – and not compositional changes of a neighborhoods as such – drive political reactions.

Before moving to the empirical section, we should re-iterate that our theoretical expectations do not result from an issue-based voter response to partisan policy supply in the domain of housing politics. We do not argue that people who face high rental market risk support the radical right because the radical right would promote the best available policy solutions for their situation or because the left has disappointed their expectations in that regard (cf. Chou & Dancygier, 2021; Cavaillé & Ferwerda, 2023). In line with the recent literature on status politics, the underlying mechanism is not so much issue-based but rather a result of a more diffuse sense of alienation and anti-elite sentiment that fuel support for radical parties that fundamentally challenge the political status quo.

In general, this does not rule out that radical right parties can strategically exploit the issue of housing. However, radical right policy supply that caters to the (ascribed) issue preferences of economically vulnerable renters is not a necessary condition for the mechanism we outline in this article. In fact, in the case studied in this article, mechanisms of issue voting are implausible because the AfD has been a strong proponent of pro-ownership and pro-market policies, an issue-specific platform similar to that of the right-liberal Free Democratic Party (FDP) (cf. Cohen, 2023). However, we acknowledge that in other political contexts, mechanisms of issue voting may augment our proposed mechanism of status threat and alienation. We discuss this in more detail in the conclusion.

## Case and Data

### The German Rental Market

**Figure 2.**
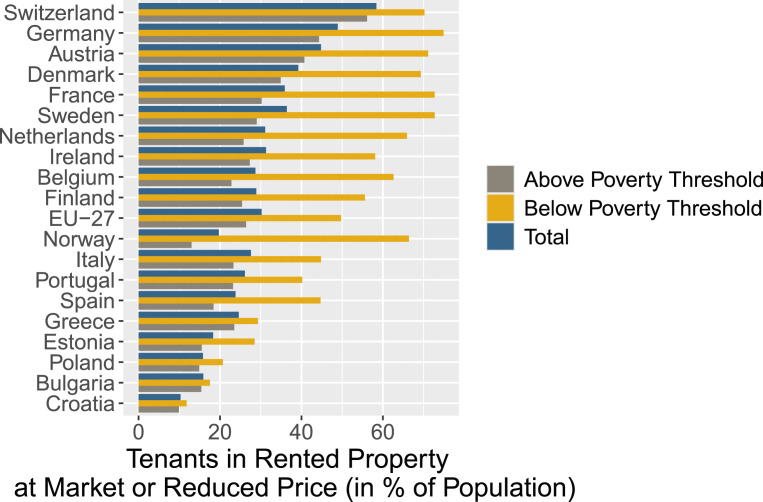
Importance of the rental market in comparative perspective (Source: Eurostat).

To test our theoretical expectations, we focus on the case of Germany, the country with the highest share of people in rental housing in the European Union. According to Eurostat data presented in Figure 2, in 2019 every second person in Germany (48.9%) lived in a rented apartment or house, compared to an EU-28 average of 30.8%. Rents account for a large share of household expenditures: on average, German tenants spend a quarter of the disposable income on rents. Both the prevalence of renting and the share of the disposable income spent on housing is socially stratified. Lower-income households live more frequently in rented as opposed to owner-occupied dwellings and spend higher shares of their income on housing. Single households and single parents spend more than a third of the disposable income on renting and this share rises to almost 50% among the poverty-vulnerable population in Germany.^3^ Given the sizable share of rents in the budgets of German households, and particularly among those with fewer economic resources, increases in local rents can pose a serious threat to tenants’ well-being and comfort.

While the German rental market has undergone drastic changes in the past two decades, these have had differential repercussion on long-term tenants and new renters due to various legal provisions. Once renters sign a regular lease, their tenancy is open-ended and, in absence of violations of the terms of contract, can only be terminated under exceptional circumstances. For existing contracts, rent installments can only be increased up to the amount of the average local rent of a comparable units in terms of size, location, and amenities every 15 months, yet by no more than 20% every three years. Therefore, increases in local rents are primarily driven by new rentals, often in conjunction with renovations and upgrades to amenities.^4^ As a result, booms in local market rents for new rentals translate into increases in rent levels for existing rentals with significant mitigation and delay.

However, increasing market rents also pose a significant threat to tenants in case they need to find a new home. On the one hand, this may be the case when landlords exercise their special right of termination upon requiring a rental unit for their own personal use. On the other hand, changes in tenants’ individual circumstances – e.g., the birth of (additional) children, grown-up children leaving the household, entering or ending a relationship – may induce the need to move to a smaller or larger dwelling. In localities with high rental market risk, such tenants will be immediately exposed to higher local rents.

Whereas two prominent policy instruments exist that allow renters to find affordable housing, neither effectively shields them from rental market risks in the long term. The first is housing allowances, which applicants, subject to meeting the eligibility criteria, are awarded for up to 12 months. Housing allowances thus offer short-term support in times of acute hardship but do not shield residents from rental market risk in the medium or long term. The second policy instrument is social housing. In short, investors receive public subsidies for the construction of new housing units under the condition that a certain share be rented at a reduced rental rate to households eligible for social housing. This obligation is, however, temporary: It usually ends 15 years after receipt of the subsidies. Thus, social housing is inherently a temporary shield against local rent price appreciation. This is exacerbated by hurdles to finding new social housing. Entitlement certificates are valid for only one year and may not be renewed if applicants fail to meet the eligibility criteria. Additionally, the social housing sector in Germany is very small in comparative perspective across Europe (IZA – Institute for the Study of Labor, Braga and Palvarini, 2013) and continues to shrink: In 2022, social housing accounted for only 1.09 million out of a total of 43.1 million residential units in Germany, a reduction by almost 50% since 2006 (DESTATIS, 2023; Deutscher Bundestag, 2023).

### Data

Our conceptualization of local rental market risk demands that we link longitudinal micro-level data with fine-grained spatial data on local rental markets. We overcome the critical empirical challenge of accurately measuring local rental price levels and changes at a granular level by using an innovative proprietary data collection: The so-called *Mietmarktmonitor* (rental market monitor), collected by the German research and consulting firm *F + B*, which provides a systematic collection of geo-referenced housing advertisements across newspapers and online market places since the early 2000s. Based on a comprehensive data set of over 27 million unique advertised rental objects, we have obtained yearly aggregates of rents and rent market indicators at the postcode level between 2005 and 2018.

Germany is divided into roughly 8,200 postcode areas, whose size strongly depends on population density. The median size of a postcode is approximately 25 sqkm, while the 2.5 percentile is 0.5 sqkm and the 97.5 percentile is 175 sqkm. Postcode areas thus capture small-scale areas that individuals usually navigate on a daily basis. The data set includes information on local market rents, local hedonic rents (i.e., modeled rents for a hypothetical reference object from a predictive model), and various indicators on the quality and quantity of the advertised objects.

Our micro-level data comes from the German Socio-Economic Panel (GSOEP). Specifically, we use a restricted-access version of the data set which is exclusively accessible for on-site use at the GSOEP Research Data Center in Berlin, Germany. Next to the extensive information on individuals’ labor market position, economic resources, and housing situation included in the standard GSOEP data, this restricted-access version includes the exact five-digit postcode of each respondents’ current place of residence. This allows us to combine the micro-level panel data from the GSOEP with our time-series cross-sectional data on local rental markets by respondents’ five-digit postcode for all survey waves from 2005 to 2018.

### Local Variation in Rental Markets

**Figure 3.**
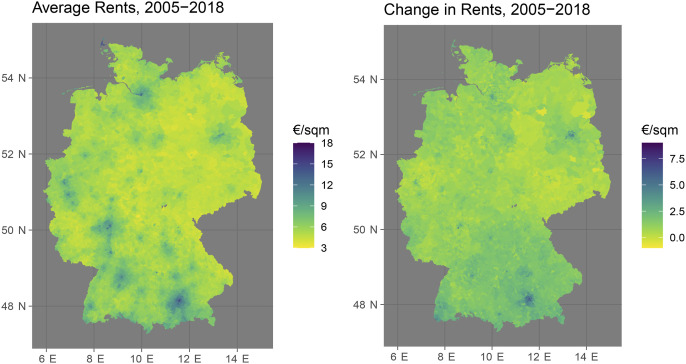
Rental market monitor data: Average levels and changes of hedonic rents at the postcode level in Germany.

Figure 3 provides an overview of the spatial distribution of postcode-level rents between 2005 and 2018. The map on the left-hand side shows the average levels of hedonic rents in €/sqm across the 14 year period whereas the right-hand side map shows the absolute change from 2005 to 2018. In the plot on the left-hand side, light-shaded areas indicate postcode areas with below-average levels while areas with above-average levels are displayed in darker colors. On the right-hand side, light colors indicate static rental markets whereas darker colors depict areas with increasing rents. As we can see on the left-hand side of the figure, the distribution of rent levels follows intuitive patterns. Between 2005 and 2018, rents have been highest in urban centers (including Munich, Berlin, Hamburg, Stuttgart, the Rhein-Main Metropolitan Region, and the Rhineland Metropolitan Region) and in popular tourist destinations (including the island of Sylt and the Alpine regions at the southern border). The right-hand side shows that this variation in rent levels correlates strongly – but far from perfectly – with the 14-year change in rents. Whereas many urban centers (including Munich and Berlin) have experienced dramatic increases in rent levels, this trend is much less pronounced or even non-existent in other West German metropolitan areas, including the Rhineland Metropolitan Region or the Ruhr Region. That said, rents have also increased significantly in many rural and suburban areas, especially in Southern Germany and in the Berlin/Brandenburg Metropolitan Area.

**Figure 4.**
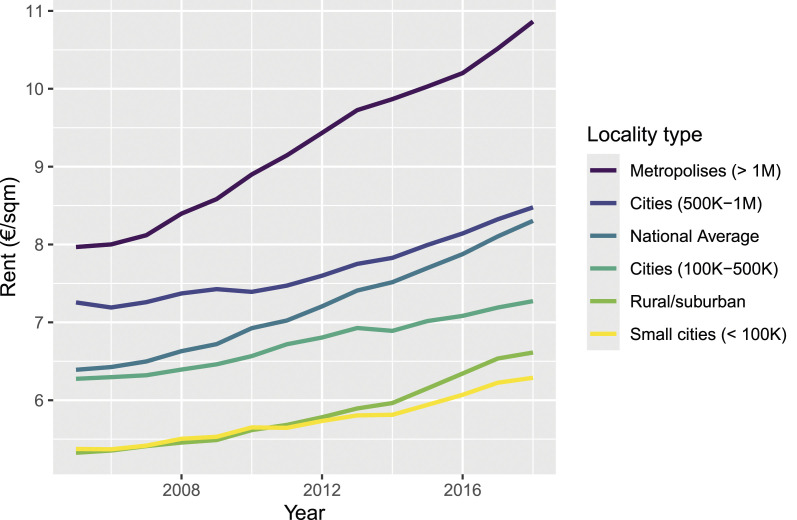
Rental market monitor data: Average trends in local rents by locality type.

Figure 4 investigates how the average 14-year trend varies across different locality types. Next to a trend line for the national average, the figure shows separate trend lines for postcode areas in rural and suburban localities (administrative counties called *Landkreise*) and urban localities (administrative counties called *Stadtkreise* and *kreisfreie Städte*, which we further distinguish by their total population). As we can see, the trend lines for rural and suburban localities and small cities with a population below 100,000 are tightly clustered and fall below the national average, where rents increased from 5.69 €/sqm in 2005 to 6.96 €/sqm in 2018. For cities with a population of 100,000 to 500,000, average rent levels are higher but the trend line runs flatter, which shows that rents in these places have, on average, grown comparatively mildly. In large cities with a population of 500,000 to 1,000,000, in contrast, the trend line starts at fairly high levels and runs parallel to the national average trend. Lastly, average rents in the German metropolises of Cologne, Munich, Hamburg, and Berlin have increased most rapidly, rising from a high starting point of approximately 8.00 €/sqm in 2005 to nearly 11.00 €/sqm in 2018 on average.

**Figure 5.**
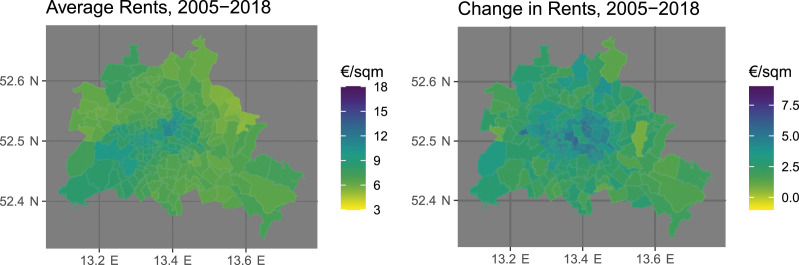
Rental market monitor data: Average levels and changes of local rents at the postcode level in Berlin.

While these overall patterns may suggest that rental market dynamics follow a regional rural-urban divide, Figure 5 zooms in on a single city – Berlin – to show that the picture is much more intricate when we take the local level into account. The figure shows astounding patterns at the postcode-level with respect to the heterogeneity in rent levels and rent developments as well as with respect to their mutual correlation. Whereas average rents are highest in the central district of Berlin-Mitte and the wealthy south-western parts of the city, prices have increased most strongly in the central districts surrounding Berlin-Mitte and also in several peripheral districts in the north and west. This highlights that even though the German rental market is broadly structured along an urban-rural divide, individual exposure to rental market risks varies considerably across neighborhoods within one and the same locality.

### The Correlates of Local Market Rents

Descriptive analyses of the correlates of local market rents, presented in Table 1, confirm a few likely expectations. We first retrieve estimates of the associations between local rents and various household-level outcomes from the GSOEP. We use a series of bivariate two-way fixed-effects models. Therefore, coefficient estimates indicate within-household associations at fixed points in time. Unsurprisingly, higher local rents correlate with higher individual rents. On the flip side, we see that local rents are associated with overall asset gains, a pattern that is much more strongly pronounced among homeowners than it is among renters. This can, in large part, be attributed to a strong correlation between market rents and owner’s net wealth from their primary residence (i.e., its market value minus mortgages). This underlines the strong discrepancy in the effects of housing market dynamics on the economic fortunes of renters in comparison to homeowners.

**Table 1.** Twoway Within-Estimates From Bivariate Regressions of Local Rents on Various Outcomes at the Level of Households and Neighborhoods.

Outcome	Level	Effect	*N*_*Obs*_	*N*_*PLZ*_
Rent/sqm (R)	Household	0.31 (0.26, 0.37)	96,993	4775
Asset wealth (R)	Household	6180.11 (401.90, 12179.96)	18,000	3560
Asset wealth (O)	Household	45339.30 (20186.18, 69846.01)	19,767	3967
Net wealth from residence (O)	Household	36910.50 (31205.02, 42775.99)	18,000	3560
Social status	Neighborhood	0.04 (0.03, 0.05)	118,922	6044
Purchasing power	Neighborhood	415.08 (371.59, 457.69)	132,283	6060

In a second set of analyses, we leverage the MICROM data available at the GSOEP Research Data Centre. MICROM is a market research company that collects various data on the composition of small-scale neighborhoods, typically just a few blocks of houses with no more than 500 residents. These neighborhoods are perfectly nested within postcode areas, which allows us to study the correlations between local rents and neighborhood characteristics. Our two-way fixed-effects estimates show positive associations with social status and purchasing power. This suggests that as local rents increase, the social composition of neighborhoods changes in that more economically affluent individuals move into the area.

## Empirical Strategy

The descriptive findings presented in the preceding section have important implications for our modeling strategy. They show that local rental market dynamics affect residential contexts by changing the *composition* of neighborhoods. This implies an important scope condition of our proposed geotropic mechanism, which emphasizes how local rental market risks threaten the established social, economic, and residential position of renters. People who have only recently moved into a neighborhood not only have weaker ties to the local area and lack a frame of reference for how the local rental market has changed compared to previous years. They may also have deliberately selected into a booming neighborhood, possibly for the very reason of its increasing popularity. This clearly sets them apart from long-term residents, who have been continuously exposed to the geotropic effect of the changing local rental market. Thus, our proposed mechanism only applies to long-term residents who have lived in a given neighborhood for some time and have experienced the changing nature of their residential environment. Therefore, we focus on individuals who have lived at their current address for at least five years.^5^

Our argument emphasizes the latent threats from changing local rental markets on the party preferences of renters. To capture this argument empirically, our quantity of interest is the effect of local market rents net of possible indirect effects on party preferences that unfold through actual household rents. Accordingly, we estimate the effect of nominal local market rents on party preferences while adjusting for renters’ actual nominal household rents (measured in €/sqm).^6^ This allows us to capture the geotropic effects of local rents while blocking portions of the effect of local rents that may potentially unfold through individuals’ pocketbooks.

To juxtapose the political effect of local market rents for renters on the one hand and owners on the other, we supplement our renter models with models we ran on a subset of respondents who are owners of their primary residence.^7^ To capture the expected dependency of local rental market risk on households’ economic resources, we interact local market rents with household income. Specifically, we use the households’ net monthly income, equivalized by the square root of the number of household members, and take the natural logarithm of this variable. In a subsequent extension, we study this interaction across regional subsets to test for heterogeneity in the proposed mechanism between rural, suburban, and urban areas.^8^

Our focus is on *within-individual effects* of changing local rent levels on party preferences. We retrieve estimates of these within-effects using a within-between model with individual-level random effects (Bell & Jones, 2015), a variant of Mundlak’s (1978) correlated-random effects estimator. In its basic form, this model yields identical effects to a model with individual-level fixed effects. Unlike the fixed effects model, however, it can be easily extended to account for additional levels of clustering through additional random effects. This is crucial for our application, because our main predictor, local market rents, is a postcode-level variable. Furthermore, we should expect notable spatial dependence among individuals from the same county or city (both of which are identical administrative categories in Germany). To account for these spatial dependencies, we extend the model to include postcode-level and county/city-level random effects.

While this modeling strategy allows us to eliminate unobserved between-individual heterogeneity in the estimation of our within-effects, our estimates remain prone to dynamic confounding: Rent price appreciation and the political developments of mainstream party decline and increasing niche party support are processes that are likely correlated, but by far not all of this correlation can be attributed to a causal relationship between the two. We therefore include year fixed effects, which absorb annual levels in both local rents and party support and thereby allow us to retrieve within-individual effects at fixed points in time. Thus, our modeling strategy vastly corresponds to a two-way fixed-effects models, but allows to take into account the clustering of units in postcode sectors and counties/cities.

We further minimize the risk of confounding by controlling for a number of possible time-varying confounders. On the one hand, these include household characteristics, such as household composition, the proportion of economically active household members, and whether a household moved in the past 12 months (within the same zip-code area). On the other hand, we control for individual-specific variables: Respondents’ labor market status and their personal contribution to the overall household income. Given the within-between formulation of our model, we include unit-demeaned versions of these variables along with their unit-means. Further information on the model specification is presented in Online Appendix F.

**Figure 6.**
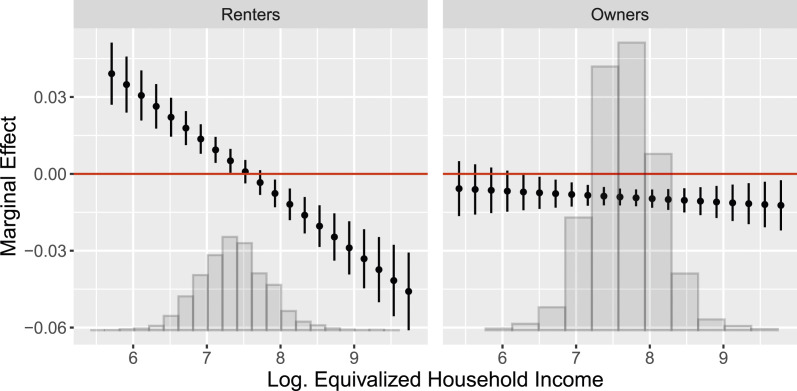
Conditional marginal effects of local market rents on the probability of AfD support as a function of logged equivalized household incomes for long-term resident renters (left) and homeowners (right). Point estimates with 95% confidence intervals. Based on the estimates reported in Table F.3 in the Online Appendix.

**Figure 7.**
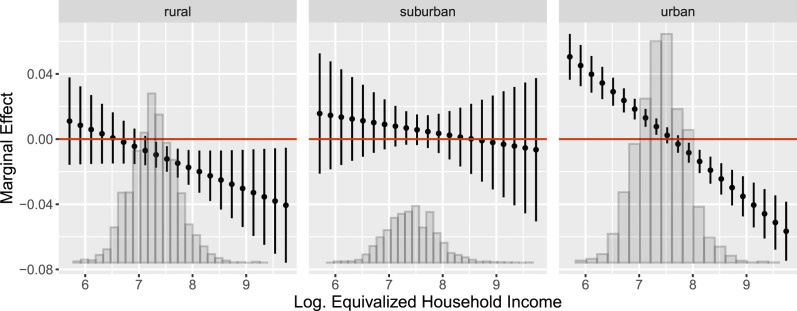
Conditional marginal effects of local market rents on renters’ probability of AfD support as a function of logged equivalized household incomes for long-term resident renters in rural (left), suburban (center), and urban (right) localities. Based on the estimates reported in Table F.4 in the Online Appendix.

Our outcome variable, party support, is a binary indicator based on the yearly item which party (if any) a respondent leans toward. Given the fairly recent entry of the AfD into the German party system, all models analyzing individual support for the AfD use a time series from 2014 to 2018. All of our models use time-varying cross-sectional sampling weights. In all of our analyses, we address the problem of missing data via multiple imputation using Amelia II (Honaker et al., 2015). We run each model on five imputed data sets and combine the estimates by first simulating the sampling distribution of the model parameters within each imputation and subsequently pooling the simulated sampling distributions across imputations. Our reported marginal effect estimates show the medians (point estimates) along with the 2.5 and 97.5 percentiles (95% confidence intervals) of these pooled simulations. In the following, we visualize these quantities of interest in Figures 6 and 7. Additionally, we selectively report the constitutive terms from which we calculate the marginal effects in Table 2. Full tabular regression output can be found in Tables F.3 and F.4 in the Online Appendix.

**Table 2.** Short Regression Table, Selectively Reporting Constitutive Terms of the Marginal Effects Presented in Figures 6 and 7. Coefficients and Simulation-Based 95% Confidence Intervals From Hierarchical Linear Within-Between Models, Estimated Across *M* = 5 Imputations. For Full Regression Output, See Tables F.3 and F.4 in the Online Appendix.

Variables	Renter Model	Owner Model	Renter Model	Owner Model
	(Overall)	(Overall)	(Locality-specific)	(Locality-specific)
Local market rent (demeaned)	0.160 [0.111, 0.208]	0.002 [−0.034, 0.038]	0.084 [−0.028, 0.197]	−0.018 [−0.073, 0.039]
Local market rent (demeaned) × household income	−0.021 [−0.028, −0.015]	−0.001 [−0.006, 0.003]	−0.013 [−0.028, 0.002]	0.001 [−0.007, 0.008]
Market rent (demeaned) × suburban locality			−0.037 [−0.236, 0.159]	0.024 [−0.072, 0.117]
Market rent (demeaned) × urban locality			0.119 [−0.008, 0.243]	0.068 [−0.016, 0.153]
Market rent (demeaned) × household income × suburban locality			0.007 [−0.019, 0.034]	−0.002 [−0.014, 0.010]
Market rent (demeaned) × household income × urban locality			−0.014 [−0.031, 0.003]	−0.009 [−0.020, 0.002]
*N*				
Observations	20,058	37,561	20,058	37,561
Individuals	7411	11,442	7411	11,442
Postcode areas	2341	2708	2341	2708
Counties/cities	390	397	390	397
Standard deviations of intercepts				
*σ*_Individuals_	0.092	0.075	0.092	0.076
*σ*_Counties/cities_	0.010	0.002	0.010	0.002
*σ*_Postcode areas_	0.033	0.037	0.033	0.036
*σ*_Observations_	0.116	0.108	0.116	0.108

## Results

### Effects of Rental Market Risk on AfD Support

We present the main results of our empirical analysis in Figure 6, which shows the marginal effect of increases in local rent levels on the probability to support the AfD conditional on household income. The results are displayed separately for renters (left) and homeowners (right). The values of equivalized household income on the horizontal axis are reported on the log-scale.^9^

The findings presented in Figure 6 are in line with our theoretical expectations. First of all, the left-hand plot confirms that rising local rental prices significantly increase the probability of supporting the radical right AfD among renters with lower household income. Over and beyond changes to respondents’ actual household rents, a hypothetical 1 €/sqm increase in local market rents increases the probability of AfD support by over four percentage points among respondents with very low incomes. In contrast, we find the opposite effect for residents with higher levels of household income. Here, a hypothetical 1 €/sqm increase in local market rents decreases the probability of AfD support by more than five percentage points. Our findings, thus, support the idea that household income strongly moderates the geotropic effect of local rent prices. For those with lower income, higher rents constitute a significant threat to their social status, which results in a higher propensity to support the radical right. For renters with higher household income, however, increasing local rents lead to less support for the radical right. We suggested that this pattern among high-income voters can be explained by a perspective of neighborhood upgrading related to the fact that this group has the financial means to enjoy the upsides of local urban development.

A direct observable implication of our argument about rental market risks is that homeowners should be shielded from the potential threat of increasing rents. The right-hand side of Figure 6 supports this expectation. For homeowners, we do not find that higher rents lead to higher support for the AfD. To the contrary, we find a negative effect, which is constant at all levels of household income. Regardless of households income streams, a hypothetical 1 €/sqm increase in local market rents decreases the probability of AfD support by roughly one percentage point. Similarly to renters with high household incomes, homeowners are beneficiaries of neighborhood upgrading, which makes them less prone to support the radical right.

This finding can also be seen against evidence that the increases in radical right voting that we find are purely driven by compositional or cultural transformations of the neighborhood such as a changing social composition of its residents or an increase in immigrants. If factors unrelated to rents were the driving force behind our findings, we should see that they affect renters and owners in the same way. In contrast, in line with our concept of rental market risk, we find that it is only low-income renters who respond to an increase in local rents with a increasing probability to vote for the radical right.

### Locality-specific Effects

Rental markets dynamics have been heterogeneously distributed across Germany: While rents have increased nearly everywhere, this development has been particularly pronounced in cities and metropolitan agglomerations (see Figures 3 and 4). We have argued that we should thus not expect the hypothesized mechanism to apply uniformly across the geo-political landscape of Germany. Instead, we expect that these developments will be particularly pronounced in high-density areas where the effects of gentrification are most tangible. In these regions, the risk of rental cost overburden is highest and the threat of being forced out of one’s long-term residential and social environment is greatest.

To test this expectation, Figure 7 shows the conditional within-effect of a 1 €/sqm increase in local rents by household income for renters living in rural (left), suburban (center), and urban (right) localities. While we find that the within-effect of rental market risk on AfD support decreases with equivalized household incomes across all three types of localities, the effect patterns differ markedly. In rural and suburban localities, rental market risk has no significant effect on low-income renters and, if anything, a moderately negative effect on renters in medium-to-high-income households. In contrast, we find that the effect pattern previously reported in Figure 6 is strongly pronounced in urban localities.

This shows that our proposed mechanism unfolds most strongly in those areas that are severely affected by rent price appreciation and where, concurrently, the proportions of individuals living in rental housing is highest. Our findings therefore uncover a dynamic mechanism that contributes to the explanation of the electoral consolidation of the AfD in ‘booming’ urban centers. In cross-sectional analyses of radical right support, these regions are typically overlooked in favor of declining regions or the static rural periphery, where radical right voting is most widespread. Our analyses thus offer new complementary evidence: Whereas structural compositional differences *between* localities predict the strong electoral appeal of the radical right in declining regions and the rural periphery, we show that even and especially in booming regions, a sizable segment of socially and economically vulnerable individuals turns to the radical right as rental market risks intensify *within* localities.

### Mechanism Evidence

Our argument posits that local market rents affect renters’ support for the radical right primarily beyond individual pocketbooks, that is, via a *geotropic mechanism*, which captures the net effect of rental market risk. Accordingly, our main analyses report the effect of local market rents (measured in €/sqm) while adjusting for household rents (equally measured in €/sqm) – a quantity known in mediation analysis as the *direct effect*. This begs the question if local rental market dynamics affect radical right support additionally, i.e., on top of our reported effect estimates, via changes in long-term residents actual household rents – a quantity typically referred to as an *indirect effect*.

We address this question empirically in Section B of the Online Appendix.^10^ Here, Figure B.3 presents estimates of the constitutive links of the indirect effect – that is, the marginal effect of square-meter local market rents on square-meter household rents, and the marginal effect of square-meter household rents on AfD support – alongside estimates of the indirect, direct, and total effects. As in the main analyses, all effects are conditional on household income and estimated from our sample of long-term resident renters. We find that, despite significant legal protection in long-term rental contracts, a hypothetical 1 €/sqm increase in local market rents results in modest increases in household rents. However, square-meter rent price increases among long-term renters do not affect the probability of AfD support, regardless of the level of household income. As a result, the indirect effect is near-zero and statistically insignificant at all levels of household income. Therefore, the total effect of local market rents is vastly dominated by our main effect of theoretical interest: The direct effect of local market rents, net of household rents.

It is important to note that this supplementary analysis is designed to explore if, on top of the effects of primary theoretical interest, effects of local square meter market rents also unfold via household’s actual rents. It is not primarily designed for addressing the question of whether the overall financial burden from actual household expenditures on rental costs drive renters towards the radical right. Other approaches that prioritize a direct measurement of how deeply rents cut into household incomes may be better suited for this specific purpose. Notably, analyzing the effects of both changes in household’s monthly rental expenditures and households’ rent loads (i.e., rental expenditures relative to household incomes), Held and Patana (2023) find significantly positive pocketbook effects on AfD support across an unrestricted sample of German renters. Our findings do not negate the existence of such pocketbook *effects* of overall rent loads. They do suggest, however, that effects of local market rents do not translate into radical support via a pocketbook *channel* among our sample of long-term resident renters.

Considering the high level of protection that long-term renters enjoy in the German rental market, this finding is plausible and fully consistent with our proposed mechanism of rental market risk. For long-term resident renters, increases in local market rents do not primarily unfold their effects through acute financial burden. Their effects unfold through the latent threat that, in the absence of sufficient financial resources, individuals may not be able to uphold their standard of living and social status at their current place of residence in the medium and long-term.

### Robustness Checks

We scrutinize the robustness of our findings in a series of additional analyses, which we report in Section D of the Online Appendix. First, we test the robustness of our findings to the inclusion of additional covariates at the local level. We focus on two popular structural explanations of far-right support: Ethnic heterogeneity and overall unemployment risk. In line with our within-between models, we add both respondent means and within-respondent demeaned variants of the corresponding measures . We measure these concepts using two variants. First, we use official annual administrative statistics, available at the county/city level, on the percentage of foreign-born residents and on the unemployment rate. The corresponding findings are reported in Figures D.6 and D.7. Secondly, to leverage more fine-grained local variation, we use small-scale neighborhood data from MICROM-SOEP. This data, provided as part of the georeferenced GSOEP data as the results of a partnership with the consumer market form MICROM, contains information at the level of small-scale neighborhood segments, which comprise about 500 households on average and are perfectly nested within postcodes. We use the proportion of ethnic majority residents and the proportion of unemployed residents in respondents’ neighborhood as additional controls and report the findings in Figures D.8 and D.9. In both versions of this robustness check, controlling for changes in the economic and ethnic composition of respondents’ place of residence does not systematically change our findings and conclusions. This shows that our findings are more than mere artifacts of compositional changes in residential environments, such as reactions to increased inter-ethnic competition, contact, or exposure. This supports our theoretical emphasis on latent economic threats from market dynamics.

Second, we scrutinize the linear interaction assumption implicit to Figures 6 and 7. We rerun our models with discrete interactions involving a tertile-binned version of respondents’ logged equivalized household income (see Hainmueller et al., 2019). The results are reported in Figures D.10 and D.11. Figure D.10 confirms the findings reported in Figure 6 above: When local rental market risk increases, long-term residents renters in low-income households become more likely to support the AfD, whereas renters in high-income households and homeowners at all levels of income become less likely to do so. Figure D.10 shows that this pattern is driven by urban localities, where renting is most common and where most renters in our sample live. Here, we see an approximately linear effect in line with that reported in Figure 7.

Third, we rerun our longitudinal analyses akin to a first difference approach, where we use respondents’ vote choice in the 2013 and 2017 German Federal Election (as retrospectively reported in the 2014 and 2018 waves of the GSOEP) in place of the annual 2014-2018 measures of respondents’ party preferences. The corresponding evidence, reported in Figures D.12 and D.13 in the Online Appendix, confirms our findings reported above. Albeit based on only two time periods, the effects from this robustness check add significant validity to our main findings. This is because in our data, voting for the AfD is more common than AfD support. As only a minority of respondents express support for any particular party, the proportion of AfD supporters remains low. In contrast, voting for the AfD in federal elections, as shown in Figure 1 above, is reported much more frequently.

Fourth, we offer two alternative specifications of the moderating local context. Instead of distinguishing rural, suburban, and urban localities, we use two use categorizations that tie the geographical context more strongly to our discussion of the economic discrepancies across regional housing markets. We use tertile-bins of local 2018 rent levels as well as 2005–2018 rent level changes therein. The corresponding findings, reported in Figures D.14 and D.15 in the Online Appendix, are fully in line with our argument. Using either of the two alternative specifications, we find no significant effects of local market risks in the bottom tertile and the strongest effects in the top tertile: In localities with the highest levels of rent prices and the highest rates of rent price appreciation, we find strong negative effects of rent price appreciation on AfD support among renters in high-income households, which are mirrored by strong positive effects among those at the lower end of the income distribution.

Fifth, we test the robustness of our findings to two more alternative model specifications. First, Figure D.20 shows the findings from a classical two-way fixed-effects model with standard errors clustered at the levels of respondents and postcode areas. This model mimics the specification of the within-respondents components of our within-between models, albeit without explicitly modeling latent similarities at higher levels of spatial clustering (due to the absence of postcode-level and county-level random intercepts). Secondly, Figure D.21 reruns our analyses with inflation-adjusted variants of local market rents, respondents’ household rents, and household incomes. The results from both robustness checks confirm the findings from our main analyses.

Sixth, we tackle alternative definitions of long-term residency. In our main analyses, we restricted the sample to residents who had lived at their current address for at least five years. Approximately 64% of homeowners, yet only 39% of renters in our full sample met this criterion. In Figures D.16 and D.17, we relax this criterion and look at medium-to-long-term residents (having lived at the current address for at least three years, which applies to 77% of homeowners and 61% of renters) as well as for all residents, irrespective of the length of their residency. While both figures support our conclusions, we see that the magnitude of the effects become weaker as we relax our sample inclusion criteria. This suggests that the effects are, indeed, most pronounced among long-term residents who have lived at the same address for five years or more. Figures D.18 and D.19 support this conclusion. They show the effects among the complementary subsets among short-term residents who moved to their current address – and thus self-selected into specific local housing markets – less than five and less than three years prior to the interview, respectively. Here, we do not find patterns comparable to those reported in Figure 6. While the effect of local market rents is decreasing in household income and statistically significant for respondents living in medium and high-income households, it is statistically insignificant among low-income respondents. Therefore, our main findings are driven by those who have been exposed to the dynamics of their local rental market for at least a few years.

### Contextualization

The preceding finding highlights heterogeneous effects across different subsets of renters and homeowners. While we characterize these subsets in terms of their housing tenure, it is likely that these subgroups are also structurally different with respect to other characteristics. To explore these, Tables E.1 and E.2 describe the subgroups in terms of various socio-structural, socio-economic, and political covariates.

One the one hand, our subgroup characterizations reveal some differences between renters and homeowners. Our renter subsamples consist of more female, younger, and urban respondents. Homeowners tend to have higher levels of educational attainment, are more often retired and less often unemployed, and have higher equivalized household incomes than renters. Homeowners showcase higher rates of having any party preference and higher support for right-wing parties, albeit not for the radical right. On the other hand, we find notable difference between the subgroups of long-term ( ≥5 years), medium-to-long-term ( ≥3 years), short-to-medium-term ( <5 years) and short-term ( <3 years) resident renters. Subsets with longer housing tenures are similarly distributed across rural, suburban, and urban localities, but tend to live in neighborhoods with slightly lower housing costs and more often in East Germany. They are, on average, older, comprise fewer respondents with tertiary education, and have higher proportions of retirees. Despite these differences, they are characterized by similar levels of household income as subsets with shorter housing tenures, and are fairly similar in their average party preferences.

Overall, these differences suggest that the subsets in which our effects of theoretical interest are most pronounced are characterized by a (mostly modest) over-representation of demographic groups that are often assumed to be averse to social change. However, it is important to note that our findings challenge the conclusion that this aversion would uniformly translate into support for the reactionary appeal of the radical right: While we find the strongest positive effect of increasing market rents on AfD support among low-income respondents within the subgroup of long-term resident renters, we also find the strongest *negative* effect among high-income respondents within this subgroup.

## Conclusion

This article studies how rental market dynamics affect political behavior. Our results suggest that rent price appreciation – a widespread phenomenon in many metropolitan and suburban areas in Europe – is a highly relevant but so far neglected source of changing electoral behavior in Europe. We contribute to a growing literature on the electoral implications of housing shortages (Adler & Ansell, 2020; Ansell et al., 2022; Cavaillé & Ferwerda, 2023; Chou & Dancygier, 2021; Held & Patana, 2023; Hilbig & Wiedemann, 2023) by studying voting behavior through the lens of rental market risk. Our empirical analysis draws on very granular rental market data to demonstrate that increases in local rent prices exert significant positive effects on individual support for the radical right. This result only applies to lower-income voters who lack the financial cushion to absorb a potential rent increase.

Our study suggests that the political consequences of housing market dynamics cannot be fully understood without considering both homeowners and renters. The classical house-price literature could take into account that rental market pressures can create significant economic risks and political shifts, particularly among lower-income renters, that may to some extent counteract the pro-system, pro-incumbent support of homeowners in the same area. This dual perspective – considering both property values and rental market conditions – offers a more complete understanding of how housing market dynamics influence political behavior. Future research should further explore these interconnected aspects to provide a comprehensive view of the political ramifications of housing economics.

The concept of rental market risk is helpful to better understand the relationship between the rental market and political behavior. It allows us to move beyond compositional differences *between* regions. Booming socio-economic regions have been shown to be the home of left-liberal voters while declining regions see higher levels of support for the radical right. Studies have successfully linked electoral behavior and political attitudes and resentment to the development of local identities and economies. These studies, however, have strongly focused on the effects of grievances resulting from structural regional decline. By highlighting how dynamic changes *within* places affect party preferences, our study provides an explanation for why we also happen to observe increasing support for the radical right in regions that are not “left behind”. By zooming in on local heterogeneity in rental market risks within localities and taking the moderating role of individual economic circumstances into account, we have provided evidence that the radical right may find considerable support even in booming regions when individuals are at risk of being overburdened and driven out by these developments.

In line with other studies of radical right support, we find that it is not experiences of acute economic hardship that drives individuals towards the radical right but the looming threat of impending economic decline in the form of latent economic risks. We do not find an effect of individuals’ *actual* household rents on support for the AfD but demonstrate the crucial role of structural changes in the *local rental market*. In response to heightened exposure to these rental market risks, which pose a significant threat to individuals’ social and economic status, voters may turn to political actors who fundamentally challenge the political status quo. Our finding that it is the radical right – and not the mainstream left – that attracts voters who face increasing economic risks in the housing market resonates with and extends recent research on status politics, which has labor market vulnerability and status anxiety to radical right support (Abou-Chadi & Kurer, 2021; Engler & Weisstanner, 2021; Gidron & Hall, 2017; Kurer, 2020). In line with those insights, we find that people exposed to profound economic risks do not necessarily turn to left-wing parties in search of economic policy solutions. Instead, the grievances that result from status threat translate into support of the populist and nativist appeal of the radical right.

While our study presents significant advancements towards better understanding the role of neighborhood effects for changes in electoral support, it can only offer a first step in this important direction. First of all, the politics of housing are of course much more far-reaching than the relationship that we analyzed in this article. We do not analyze policy supply and party platforms in terms of housing. We have thus not taken into account parties' specific policy positions on this issue but have focused on economic risks as a driver of radical right support. For our argument, it is not necessary that the radical right caters to the economic interests of economically vulnerable renters in order for rent increases to lead to more radical right support, and such mechanisms are, in fact, implausible in Germany due to the AfD’s staunchly right-wing positions on housing policy. However, in general, such mechanisms of issue voting may well augment our proposed mechanism. While radical right parties in Europe continue to focus primarily on issues like immigration, crime, and gender, some radical right parties, as in the Netherlands and Portugal, have recently embraced the issue of housing shortages. Geert Wilders and his PVV, for example, argued forcefully that immigration is a main source of housing shortages, thus trying to frame this issue in a way that is beneficial to the radical right. Our study provides an indication that this might, indeed, be a successful strategy. Future research should therefore incorporate a perspective in which parties have more agency and study how parties’ programmatic strategies can shape political competition around the issues of housing policy.

Moreover, we want to note that despite a vast literature in urban and regional sociology that study phenomena such as gentrification, there is very little exchange between this literature and political science research on electoral behavior. We hope that our work will inspire future studies to turn towards different aspects of changing social, economic, and cultural contexts within neighborhoods, and to study their downstream consequences on political preferences, political behavior, and electoral change.

## Supplemental Material

Supplemental Material - Rental Market Risk and Radical Right Support Supplemental Material for Rental Market Risk and Radical Right Support by Tarik Abou-Chadi, Denis Cohen, and Thomas Kurer in Comparative Political Studies

## Data Availability

Replication files are available in the CPS Dataverse (Abou-Chadi, Cohen and Kurer, 2024).

## Notes

1. Numbers based on a representative, nationwide survey that has looked at the concerns of the population for 31 years. https://www.die-aengste-der-deutschen.de/.

2. A core assumption underlying our conceptualization and measurement of rental market risk in terms of objective local rental market dynamics is that local residents actually perceive and process such information. Empirical evidence on the relationship between objective rental market dynamics and subjective perceptions thereof, presented in Section C of the Online Appendix, supports this assumption.

3. Destatis Share of housing costs and housing costs overburden. URL: https://www.destatis.de/EN/Themes/Society-Environment/Housing/Tables/eurostat-share-housing-costs-silc.html.

4. While federal legislation to cap increases in rent prices upon rentals to new tenants has been in place since 2015 (*Mietpreisbremse*), it applies only in few municipalities with excessive growth rates and many units (specifically, new dwellings) are exempt from the regulation. Its overall effectiveness for reducing local rents is questionable as negative effects for regulated units are accompanied by positive effects for unregulated units (Mense et al., 2023).

5. We discuss alternative definitions of long-term residency as part of the robustness checks below.

6. We forgo annual inflation-adjustments to market rents, household rents, and respondent incomes because our models, as detailed below, include year fixed effects. These absorb annual baseline differences in all predictors.

7. As we can only observe household rents for renters, we can control for actual household rents in our renter models only. Our owner models do not include this variable.

8. Our classification is a simplified version of a municipal-level typology of residential-structural areas presented by the *Federal Office for Building and Regional Planning*. We define so-called “core cities” as urban, municipalities in high and medium-density counties within broader urbanized and agglomeration areas as sub-urban, and municipalities within rural countries as rural.

9. This means that the farther we move to the right of the scale, the farther spread out the values of the underlying true scale become: The minimum value of 5 corresponds to a monthly net household income of 150€, the center of the scale at 7.5 corresponds to approximately 1800€, and the maximum value of 10 corresponds to a monthly net income of 22,000€.

10. We note that decomposing total effects into direct and indirect effects constitutes a challenge due to the stringency of the sequential ignorability assumption (cf. Imai, Keele and Tingley, 2010). We address this issue in greater detail in the Online Appendix.
