# Supplementary material for: Rental Market Risk and Radical Right Support
Source: Comp Polit Stud. 2024 Dec 8;58(13):2866–901. doi: 10.1177/00104140241306963 (PMC12404525; doi:10.1177/00104140241306963)
Supplement: Supplemental Material - Rental Market Risk and Radical Right Support [file sj-pdf-1-cps-10.1177_00104140241306963.pdf]

Online Supplementary Material:
Rental Market Risk and Radical Right Support

Tarik Abou-Chadi[†] Denis Cohen[*] Thomas Kurer[‡]

[†]University of Oxford. tarik.abou-chadi@politics.ox.ac.uk
[*]University of Mannheim. denis.cohen@uni-mannheim.de
[‡]University of Zurich. kurer@ipz.uzh.ch

Online Supplementary Material

A. The German housing market

A.1. Correlation between 2018 market rents levels and 2005-2018 market rent changes

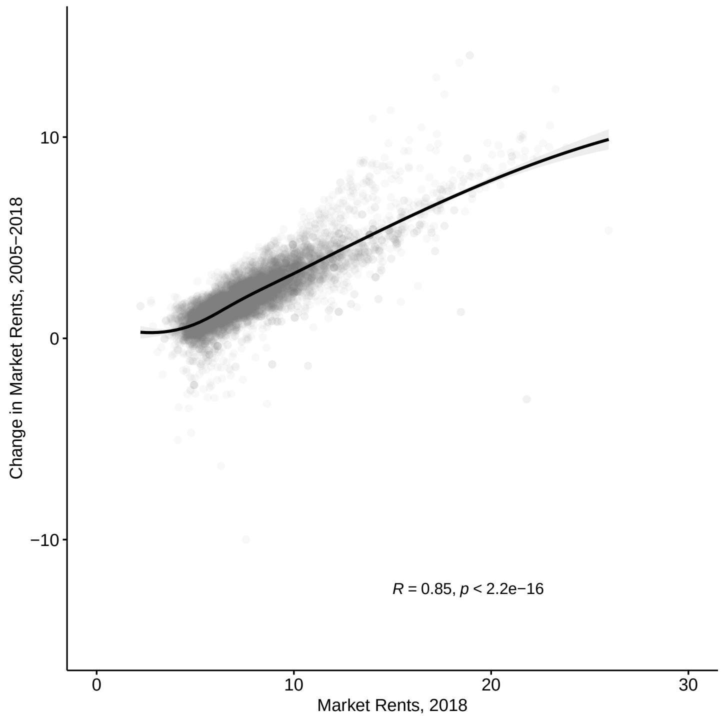

Figure A.1: LOESS estimate of the postcode-level relationship between 2018 market rents (x-axis) and 2005-2018 changes in market rents (y-axis). R gives the Pearson correlation coefficient. Based on F+B Rental Market Monitor data.

.

A.2. Correlation between market rents and property purchase prices

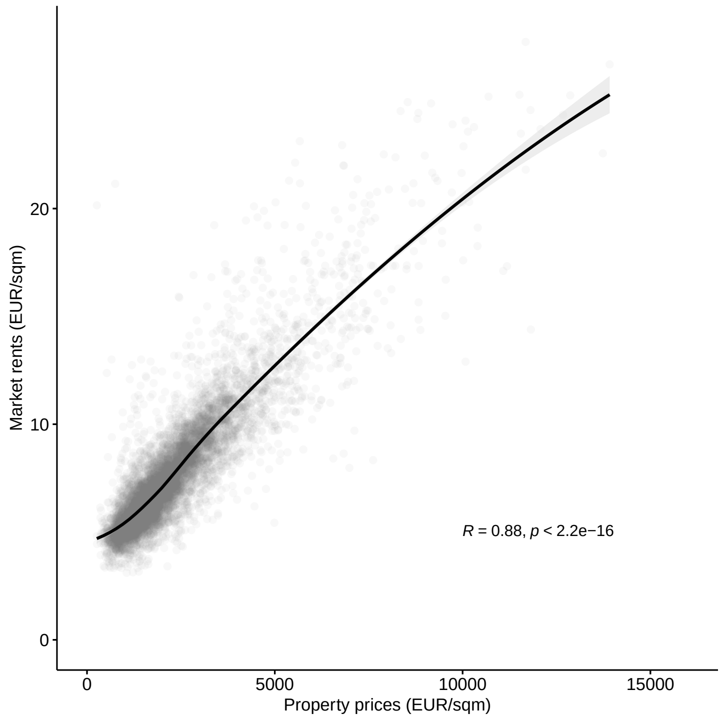

Figure A.2: LOESS estimate of the postcode-level relationship between market prices for property purchases (x-axis) and market rents (y-axis). R gives the Pearson correlation coefficient. Based on RWI-GEO-RED data (RWI; ImmobilienScout24, 2021a,b)

.

B. Mechanism evidence

B.1. Mediation analysis

The results presented in the main text lend strong support to the presence of a direct effect, thus supporting our argument regarding the presence of an income-dependent geotropic effect of rental market risk. To assess if this geotropic effect is augmented by additional effects that unfold via long-term renters' household rents, this supplementary analysis seeks to disaggregate the total effect of local market rents into direct effects that unfolds over and beyond household rents (which is the quantity of interest presented in the main text) and potential additional indirect effects that unfold through increases in households' actual monthly rents.

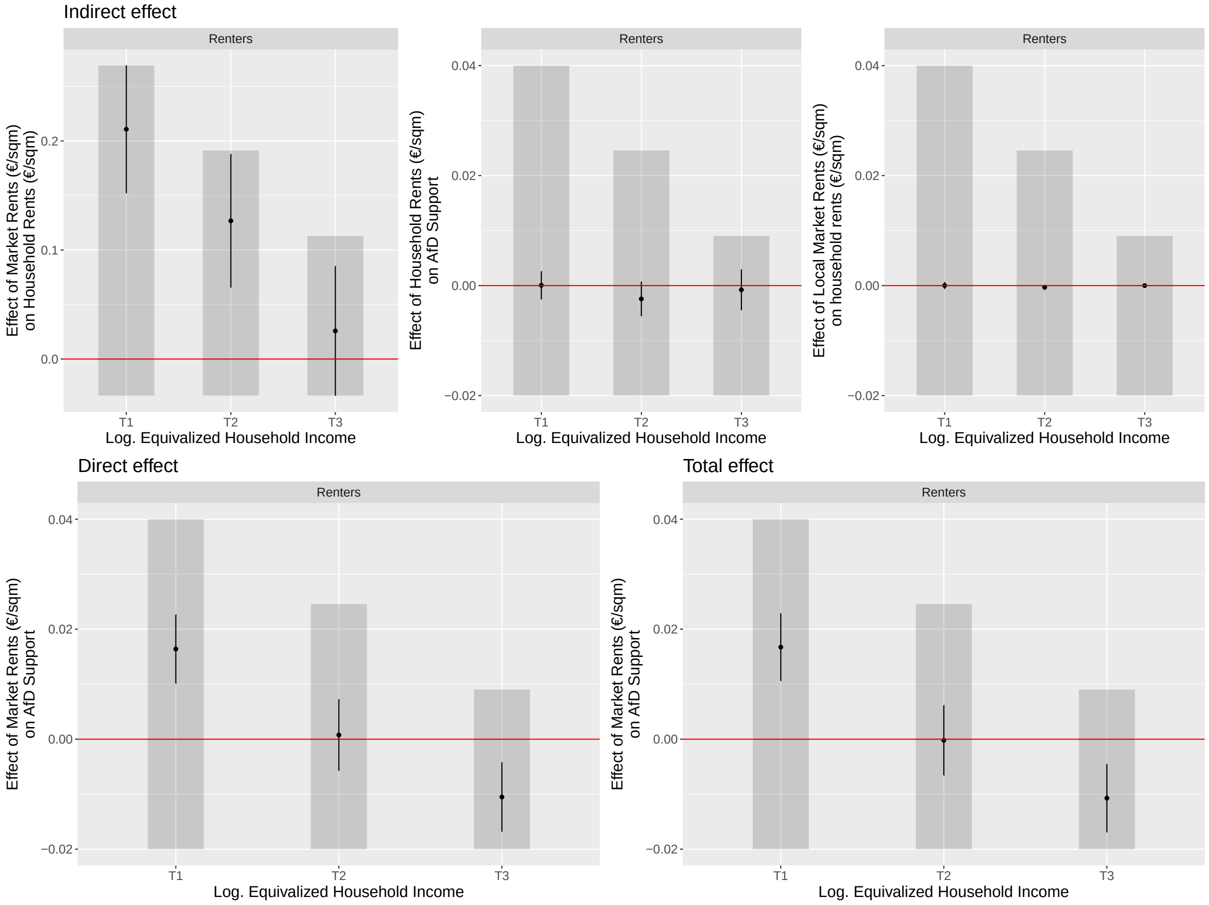

Figure B.3: Effect estimates and 95% confidence intervals (by tertiles of household income). Top left: Marginal effect of local market rents (in € /sqm) on actual household rents (in € /sqm). Top center: Marginal effect of actual household rents (in € /sqm) on AfD support (on the probability scale). Top right: Average indirect effect of local market rents on AfD support that unfolds via household rents. Bottom left Direct effect of local market rents on AfD support (i.e., net effect after adjusting for household rents). Bottom right: Total effect of local market rents on AfD support (i.e., gross effect without adjustment for household rents.

The corresponding evidence is presented in Fig. B.3. To ensure sufficient compatibility with our sensitivity analyses, which, as we detail below, require a different estimation framework than that used

in our main analyses, Fig. B.3 uses within-estimates from hierarchical within-between models with a tertile-binned moderator instead of the continuous linear interaction reported in the main text. This specification is analogous to that presented in our robustness check in Fig. D.10.

In the top panel of Fig. B.3, we present estimates of the marginal effect of local market rents (the focal "treatment" variable) on household rents (the mediator), of household rents (the mediator) on party preferences (the outcome), as well as the combined indirect effect, which, given our use of hierarchical linear models, we compute by the product method. As we can see in the first plot, even in our sample of long-term renters in the 2014-2018 period, there are significant and sizeable effects of square-meter market rent increases on square-meter household rents among low-income and medium-income respondents. A 1 €/sqm increase in local market rents results in a 0.21 €/sqm increase in household rents of low-income households, a 0.12 /sqm increase among medium-income households, and a (statistically insignificant) 0.03 €/sqm increase among high-income households. On the one hand, these effects shows how strongly long-term renters on existing contracts are shielded from immediate financial repercussions of market rents. Household rents underperform market rents much more strongly in this subgroup than in the general renter population (including new arrivees), where we estimated an effect of 0.34 €/sqm as reported in Table 1 in the main text. On the other hand, the results show that long-term contracts are not fully shielded from market dynamics. The concentration of the effects among low-income and medium-income household reflects that, in the period studied here, relative increases in rent prices were most pronounced in previously low-rent, low-income areas (Baldenius, Kohl and Schularick, 2020).

However, the results reported in the second plot of the top row of Fig. B.3 show that square-meter rent price increases among long-term renters do not seem to affect the probability of AfD support. This finding holds at all levels of household income. As a result, the indirect effect, reported on the third plot, is near-zero and statistically insignificant at all levels of household income. This also means that the total effect, reported in the bottom right of Fig. B.3, is vastly dominated by our main effect of theoretical interest, i.e., the direct effect shown to its left. This supports the relevance of our proposed mechanism: For long-term resident renters in the heavily protected German rental market, increases in local market rents hardly unfold their effects per acute financial burden, but instead through the latent rental market risks that, in the absence of sufficient financial resources, threatens individuals ability to uphold their standard of living and social status in the medium and long-term.

B.2. Sensitivity analysis

Disaggregating total effects into direct and indirect effects constitutes a challenge – especially in observational research – because of the stringency of the sequential ignorability assumption (cf. Imai, Keele and Tingley, 2010). In the context of our analyses, this assumption stipulates that two assumptions of conditional independence must hold jointly. *First*, conditional on observable confounders, local market rents must be statistically independent of the potential outcomes in both household rents and

AfD support. *Secondly*, conditional on observable confounders and local market rents, household rents must be statistically independent of the potential outcomes in AfD support. Neither of these conditional independence assumptions is ever testable or refutable. Additionally, in observational research, the first assumption cannot be satisfied by design via randomized treatment assignment. Therefore, when engaging in observational research, researchers must seek to meet these assumptions by finding credible strategies for reducing the risk of confounding, which is primarily achieved by conditioning on relevant observable confounders.

As explained in the main text, we have carefully designed our model specifications such that the risk of confounding by combining three strategies for conditioning on observables. First, we eliminate unit heterogeneity (and, thus, the all time-invariant confounders) by estimating within-respondent effects per our within-between-models. Secondly, we stratify our analyses by relevant characteristics, namely homeownership (by separating renter and homeowner subsets) and housing tenure (by subsetting the analyses to long-term residents). Lastly, we use covariate adjustment to control for a host of likely time-varying confounders. Additionally, we note that central components of the findings reported above – namely the presence of a statistically positive total effect of local market rents on AfD support among low-income long-term renters, and the absence of a corresponding significant effect of household rents – rest on standard (i.e., non-sequential) assumptions of conditional independence.

That said, seeing as both assumptions must jointly hold for credibly disaggregating the total effect into direct and indirect effects, we also conduct sensitivity analyses as proposed in Imai, Keele and Tingley (2010). In general, sensitivity analyses are theoretical exercises that help us determine help us determine how large a violation of the identifying assumption would be required to invalidate the substantive conclusions we draw from analyses that invoke said assumption. In the specific context of mediation analysis, the sensitivity analysis proposed in Imai, Keele and Tingley (2010) scrutinizes a key aspect of the sequential ignorability assumption. In the context of our research, the analysis focuses on the hypothetical presence of unobserved confounders that jointly affect the model used for estimating the effect of market rents of household rents (as displayed in the top left of Fig. B.3) and the model used for estimating the effect of market rents and household rents on AfD support (as displayed in the top center and bottom left of the same Figure). It quantifies the strength of such hypothetical confounders per a correlation parameter, ρ, that denotes the dependence between the error terms of both models. The sensitivity analysis, then, shows how the estimates of the direct and indirect effects would be biased if ρ took on any given non-zero value.

We implement our sensitivity analyses using the mediation package (see Imai et al., 2010). However, some features of our main models cannot be accommodated in the package. This pertains to our use of hierarchical linear within-between models and the continuous effect moderation by household income. Therefore, we implement OLS models that mimick the logic of the within-between models with discrete income interactions as reported above. Towards this end, we stratify our OLS models by tertiles of average household income such that we can conduct separate sensitivity analyses within each tertile. We then implement a within-estimator in the OLS framework for each terile-based sub-

set by manually demeaning both outcome and predictor variables and downward adjusting the model degrees of freedom by the number of (de facto) unit fixed effects. We use this framework to estimate both the effect of market rents of household rents as well as the effect of market rents and household rents on AfD support within each tertile (while adjusting for all time-varying confounders listed in the main text), and perform sensitivity analyses for both direct and indirect effects within each tertile.

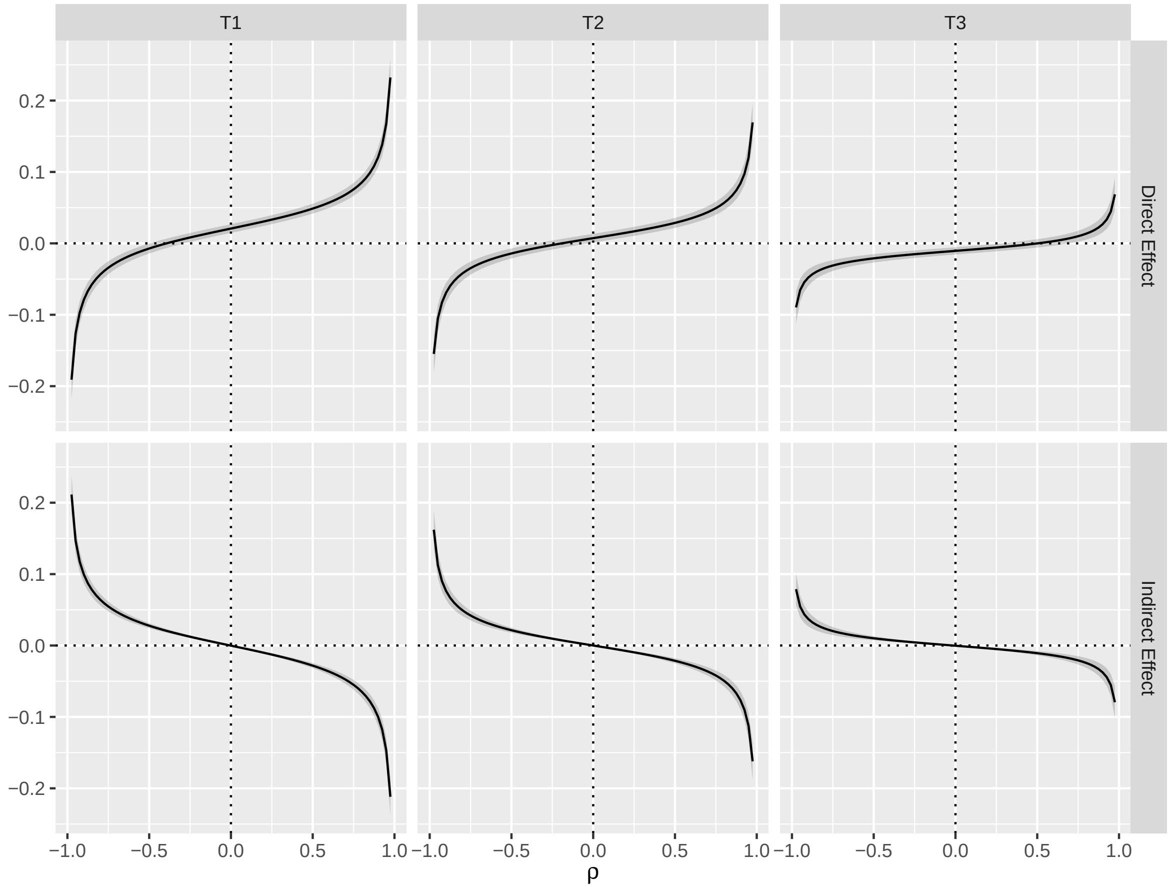

Figure B.4: Sensitivity analysis: Effect estimates and 95% confidence intervals (by tertiles of household income). Top row: Sensitivity analysis for the direct effect as function of ρ. Bottom row: Sensitivity analysis for the indirect effect as function of ρ.

The results are shown in Fig. B.4. The top panel shows the sensitivity curves for the direct effect estimates within each income-based tertile, whereas the lower panel shows the same for the indirect effect estimates. At $\rho = 0$, each plot reports the estimates produced by our adapted estimation procedure under the assumption of no confounders jointly affecting the two constitutive models. As we can see, the estimates strongly resemble those produced using our original estimation procedure, as report in Fig. B.3 above, in terms of directionality, magnitude, and uncertainty. As before, we find no significant indirect effects, a significantly positive direct effect among low-income respondents (T1), and a significantly negative direct effect among high-income respondents (T3).

As we can see from the sensitivity curves, negative values of ρ (i.e., negative hypothetical error correlations) would depress the magnitude of the effects, whereas high values would increase their magnitude. While deviations from the original estimates become large at extreme (and, thus, unlikely)

values of ρ, we find that our substantive conclusions hold up to sizable values of ρ: We would no longer find a significantly positive direct effect of market rents among low-income renters if the ρ fell below values of approximately -0.3 (i.e., in case we missed important time-varying confounders that positively affect household rents and negatively affect AfD support, or vice versa). Likewise, we would fail to find a significantly negative direct effect among high-income renters if ρ exceeded values of about $+0.4$ (i.e., in case we missed time-varying confounders that affect household rents and AfD in the same direction). Beyond these values, we would have to conclude that our the total effect of local market rents (as shown in the bottom right of Fig. B.3) unfolded through household rents as opposed to the hypothesized channel of rental market risks. While violations of the identifying assumption are always non-refutable, we believe that our principled approach outlined above renders violation of such magnitude unlikely.

C. Subjective perceptions of local rental market dynamics

A core assumption underlying our measurement and estimation of rental market risk as the net effect of local market rents while adjusting for individual household rents is that local residents register local housing market dynamics and perceive them as a threat. Such subjective perceptions of local rental markets are not available in our primary data, the GSOEP. However, we leverage a second data set, an original survey georeferenced at the postcode-level, that was fielded in Germany in late 2020 (Abou-Chadi et al., 2024).

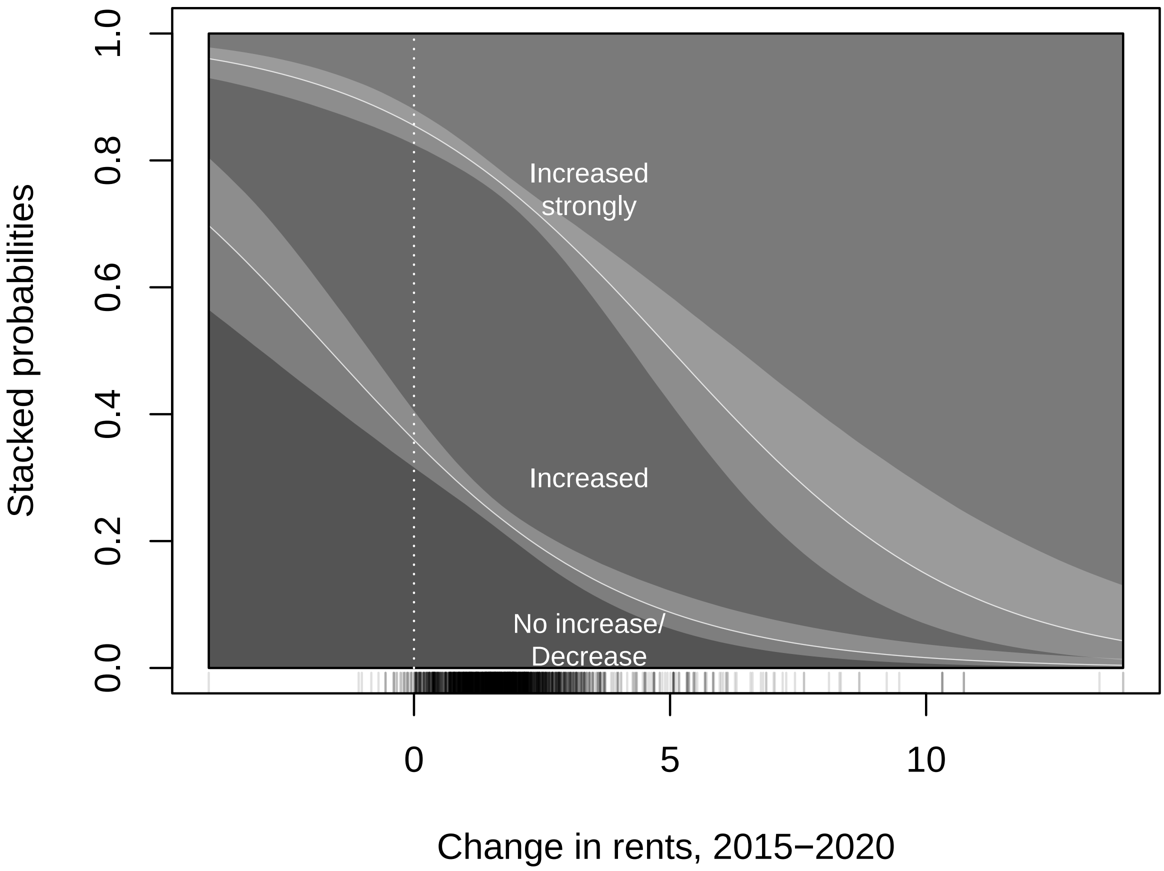

Figure C.5: Subjective perceptions of five-year changes in local rental market prices as a function of postcode-level market rents (in € /sqm). Stacked probabilities and 95% confidence intervals. The dark-shaded bottom area indicated the probability that rents were perceived to have decreased or stayed the same; the medium-dark area at the center indicates the probability that rents increased somewhat; and the light-shaded top area indicates the probability that rents increased strongly.

This survey includes an item asking *"In your opinion, how have rental costs in your current neighborhood developed over the last 5 years?"*. Responses were recorded on a five-point Likert scale ranging from "Strongly decreased" to "Strongly increased", with a neutral category "Stayed about the same" in the middle. Out of N=1,925 valid responses, only six respondents' stated that rents in their neighborhood had (strongly) decreased. 440 stated that rents had stayed about the same, 987 stated they had increased, and 492 stated they had strongly increased.

Given the sparsity of respondents who believe that rents had decreased (which accurately reflects the low percentage of postcode areas in which nominal rents had factually decreased between 2015 and 2020), we collapse the lowest three categories and run an ordered logistic regression of the trichotomous indicator of rent change perceptions on actual 2015-2020 changes in postcode-level market rents, with standard errors clustered at the postcode level.

Fig. C.5 displays the results in a stacked probability plot, with the predictor, actual changes in nominal market rents, along the horizontal x-axis. The dark-shaded area underneath the lower white line shows the proportion of respondents who believe that rents remained stable or decreased; the medium-dark area in between the two white lines shows the proportion of respondents who believe that rents increased somewhat; and the light-shaded area above the upper white line shows the proportion of respondents who believe that local rents increased strongly. As we can see, actual changes in market rents strongly predict subjective perceptions. This supports our assumption that local residents accurately perceive housing market dynamics in their residential environment.

D. Robustness checks

D.1. Addition of contextual covariates: Local unemployment rate and ethnic heterogeneity

D.1.1. Version 1: Using official statistics at the county/city level

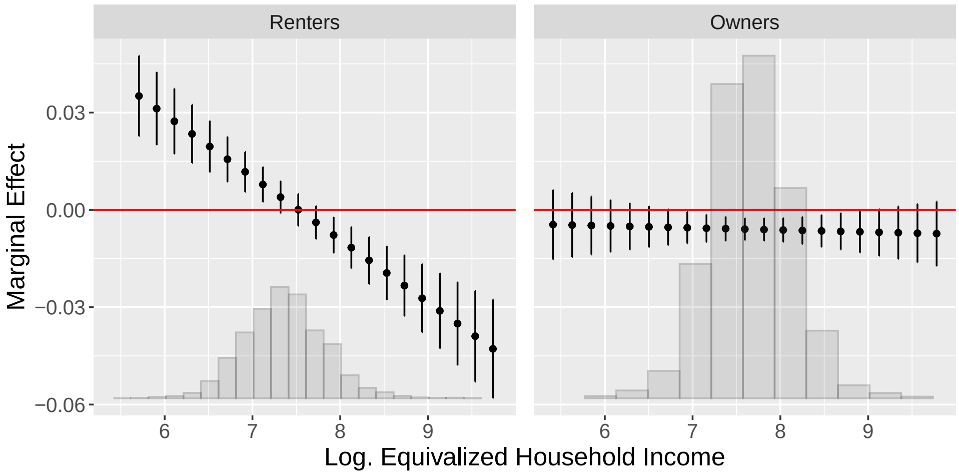

Figure D.6: Conditional marginal effects of local market rents on the probability of AfD support as a function of logged equivalized household incomes for long-term resident renters (left) and homeowners (right). Point estimates with 95% confidence intervals. Note: In addition to the covariates included in the main models, this model includes within and between effects for county-level unemployment rates and the county-level percentage of foreign-born residents.

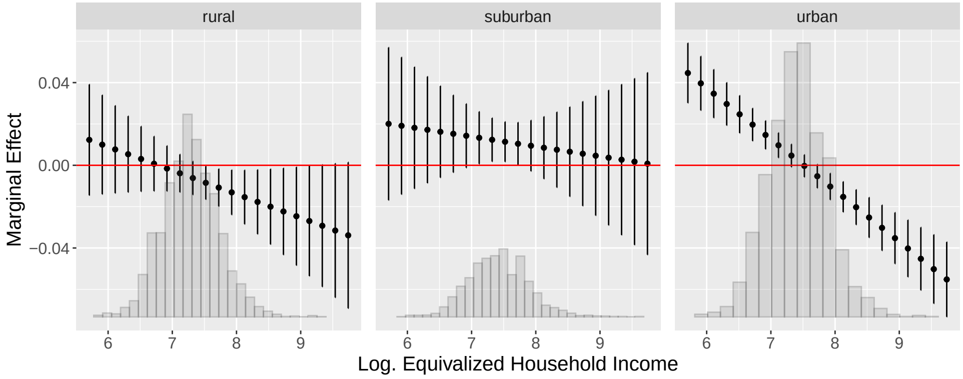

Figure D.7: Conditional marginal effects of local market rents on the probability of AfD support as a function of logged equivalized household incomes for long-term resident renters in rural (left), suburban (center), and urban (right) localities. Point estimates with 95% confidence intervals. Note: In addition to the covariates included in the main models, this model includes within and between effects for county-level unemployment rates and the county-level percentage of foreign-born residents.

D.1.2. Version 2: Using microm data at the neighborhood level

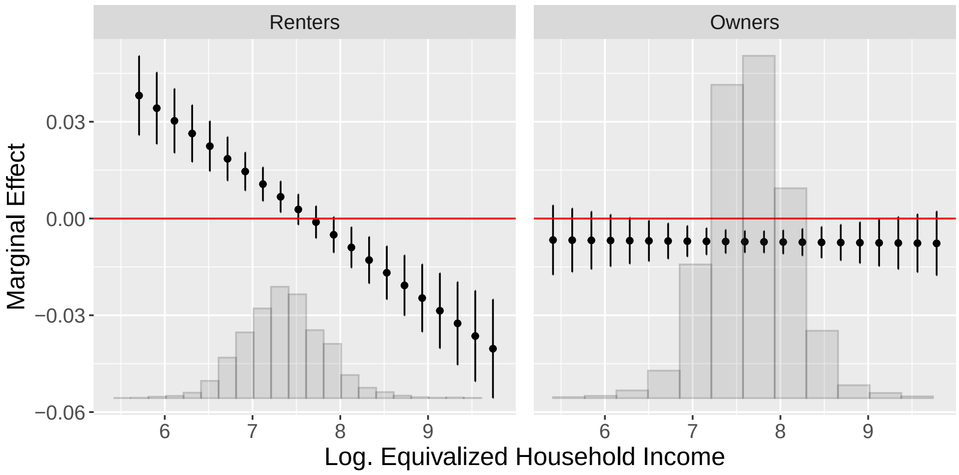

Figure D.8: Conditional marginal effects of local market rents on the probability of AfD support as a function of logged equivalized household incomes for long-term resident renters (left) and homeowners (right). Point estimates with 95% confidence intervals. Note: In addition to the covariates included in the main models, this model includes within and between effects for neighborhood-level unemployment rates and the neighborhood-level proportion of ethnic majority residents, both based on SOEP-microm data.

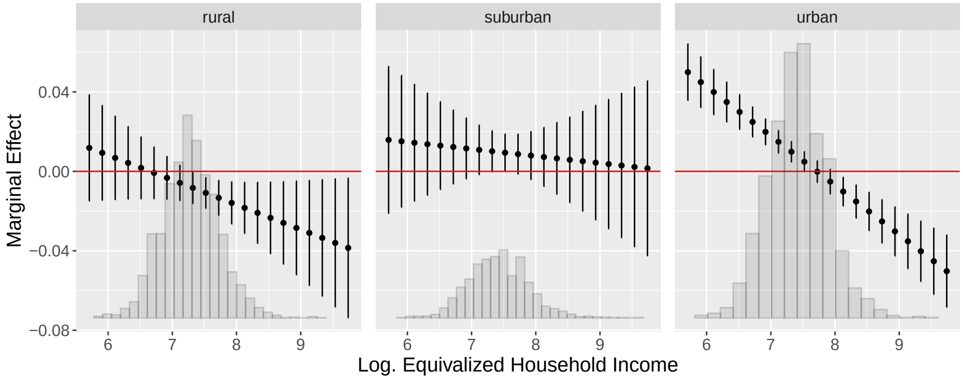

Figure D.9: Conditional marginal effects of local market rents on the probability of AfD support as a function of logged equivalized household incomes for long-term resident renters in rural (left), suburban (center), and urban (right) localities. Point estimates with 95% confidence intervals. Note: In addition to the covariates included in the main models, this model includes within and between effects for neighborhood-level unemployment rates and the neighborhood-level proportion of ethnic majority residents, both based on SOEP-microm data.

D.2. Testing the linear interaction assumption: Tertile-binned household income

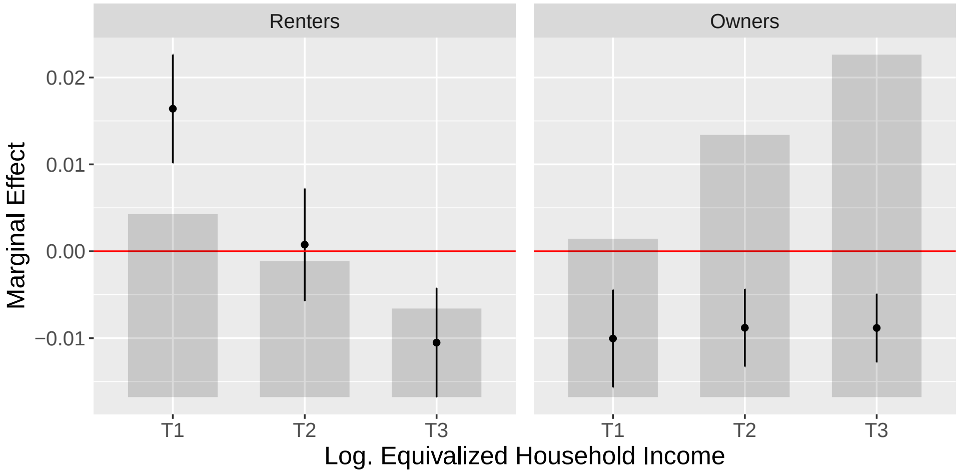

Figure D.10: Conditional marginal effects of local market rents on the probability of AfD support as a function of tertile-binned logged equivalized household incomes for long-term resident renters (left) and homeowners (right). Point estimates with 95% confidence intervals.

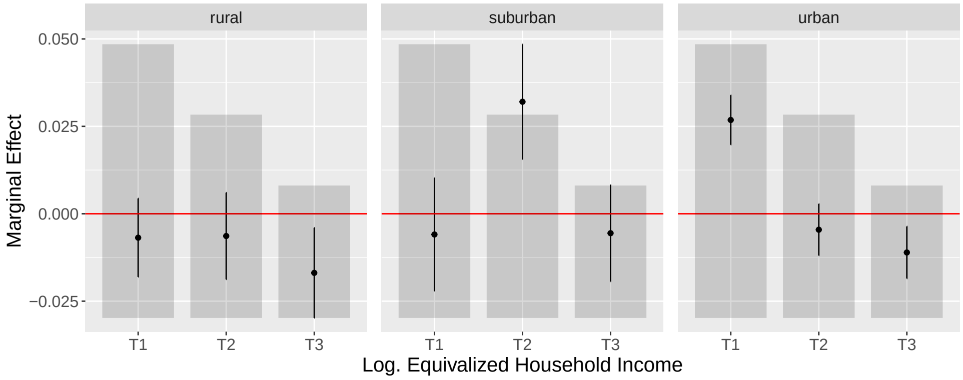

Figure D.11: Conditional marginal effects of local market rents on the probability of AfD support as a function of tertile-binned logged equivalized household incomes for long-term resident renters in rural (left), suburban (center), and urban (right) localities. Point estimates with 95% confidence intervals.

D.3. Alternative measurement of the outcome: AfD vote choices in 2014 and 2018

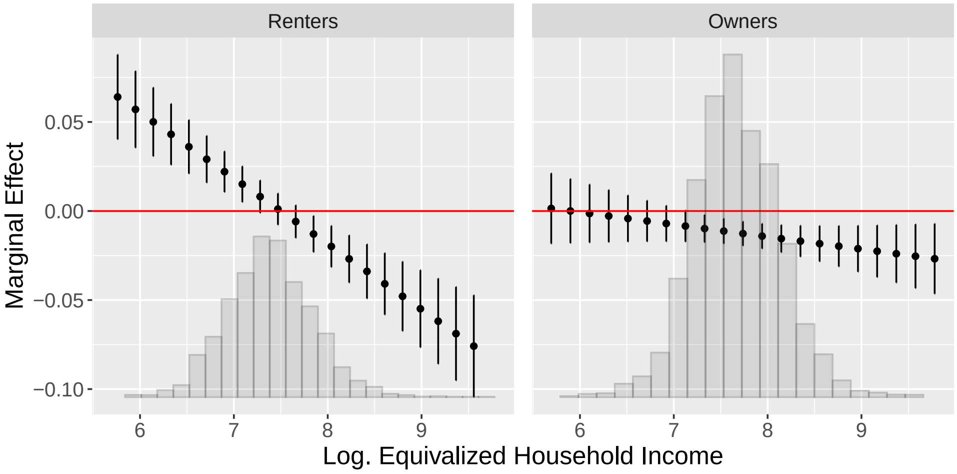

Figure D.12: Conditional marginal effects of local market rents on the probability of AfD votes as a function of logged equivalized household incomes for long-term resident renters (left) and homeowners (right). Point estimates with 95% confidence intervals.

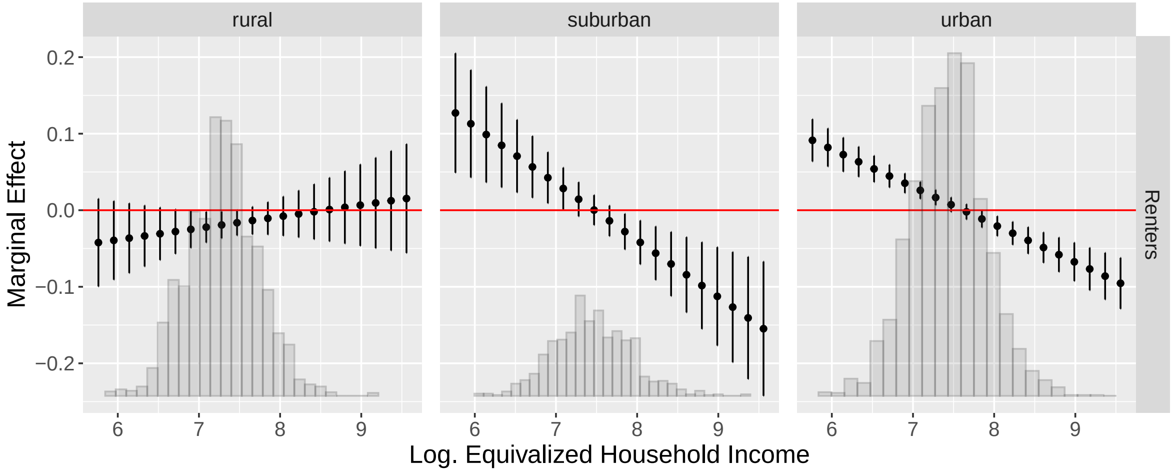

Figure D.13: Conditional marginal effects of local market rents on renters' probability of AfD votes as a function of logged equivalized household incomes for long-term resident renters in rural (left), suburban (center), and urban (right) localities.

D.4. Alternative measurement of the local context

D.4.1. Tertiles of local 2017 rent levels

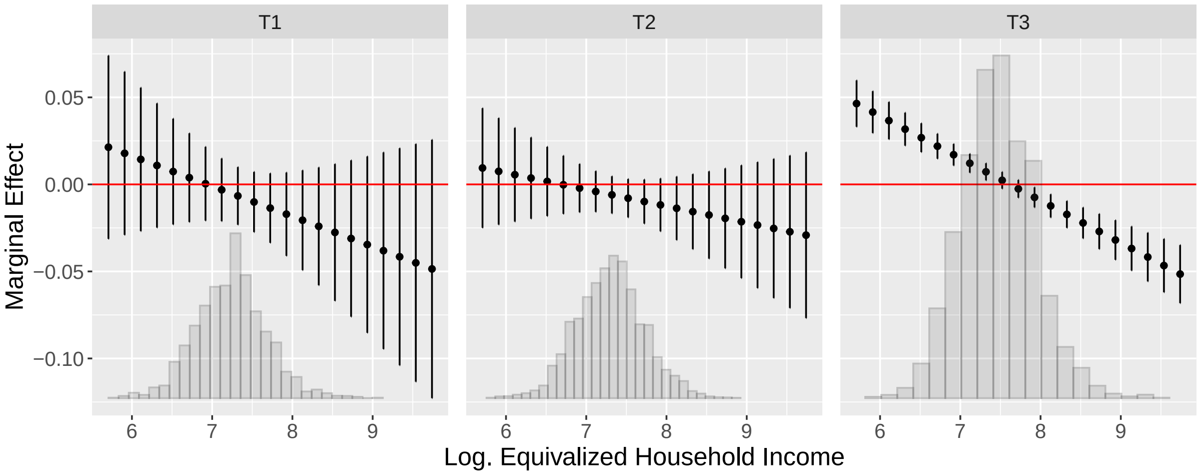

Figure D.14: Conditional marginal effects of local market rents on renters' probability of AfD support as a function of logged equivalized household incomes for long-term resident renters in low-rent (left), medium-rent (center), and high-rent (right) localities.

D.4.2. Tertiles of local 2005-2018 rent level changes

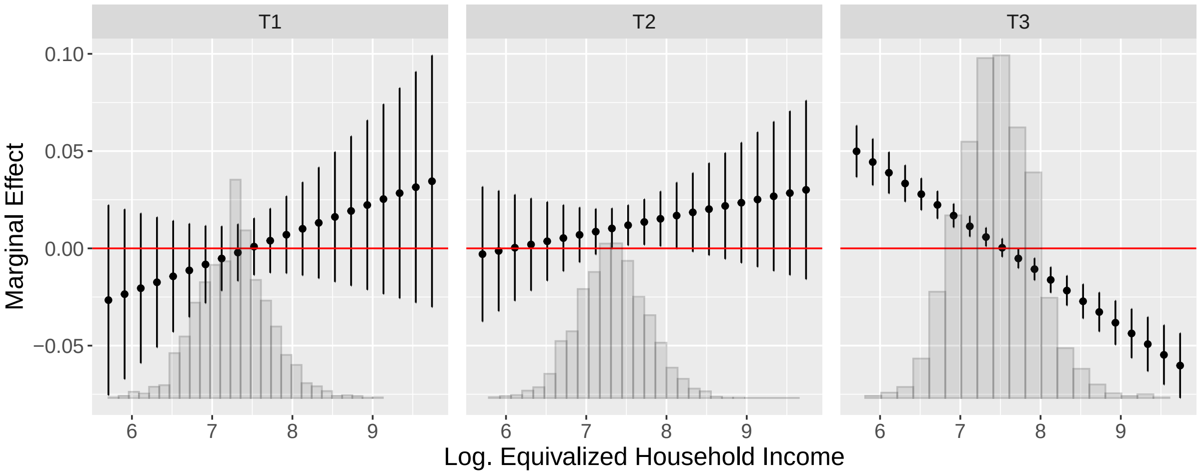

Figure D.15: Conditional marginal effects of local market rents on renters' probability of AfD support as a function of logged equivalized household incomes for long-term resident renters in localities with low (left), medium (center), and high (right) levels of rent price appreciation.

D.5. Robustness: Length of residency

The subsets can be interpreted as follows:

- *All*: All residents
- <3 *years at address*: Residents who moved to their current address within three years of the interview date
- <5 *years at address*: Residents who moved to their current address within five years of the interview date
- ≥ 3 *years at address*: Residents who moved to their current address at least three years before the interview date
- ≥ 5 *years at address*: Residents who moved to their current address at least five years before the interview date

D.5.1. All current residents

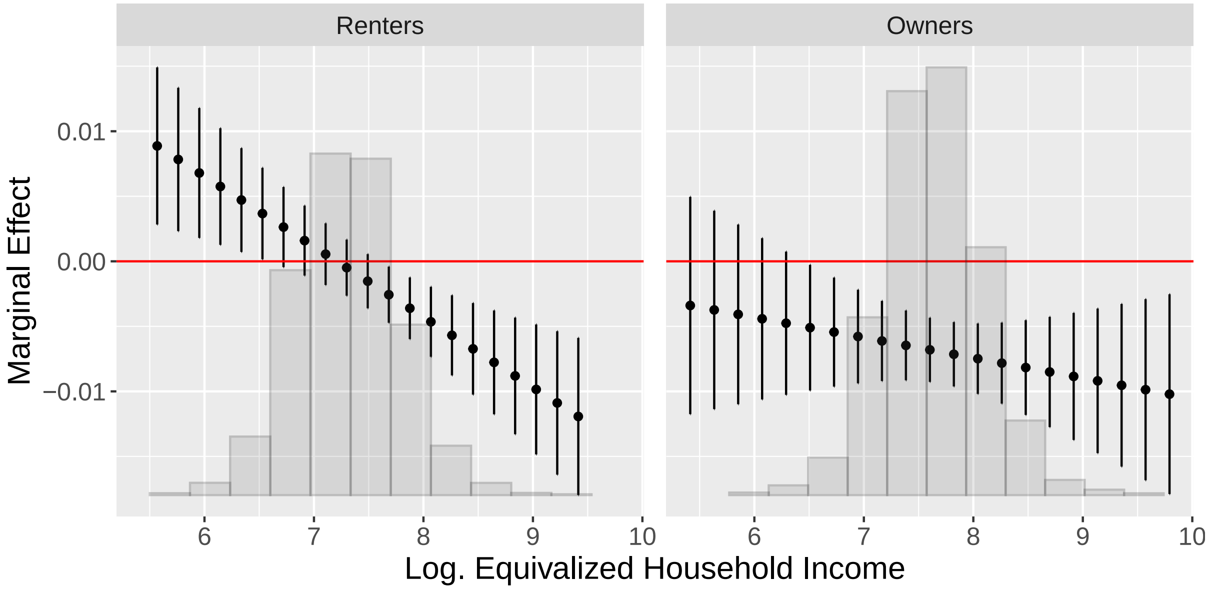

Figure D.16: Conditional marginal effects of local market rents on renters' probability of AfD support as a function of logged equivalized household incomes for renters (left) and owners (right) without minimum residency length.

D.5.2. Medium-to-long-term residents: ≥ 3 years at address

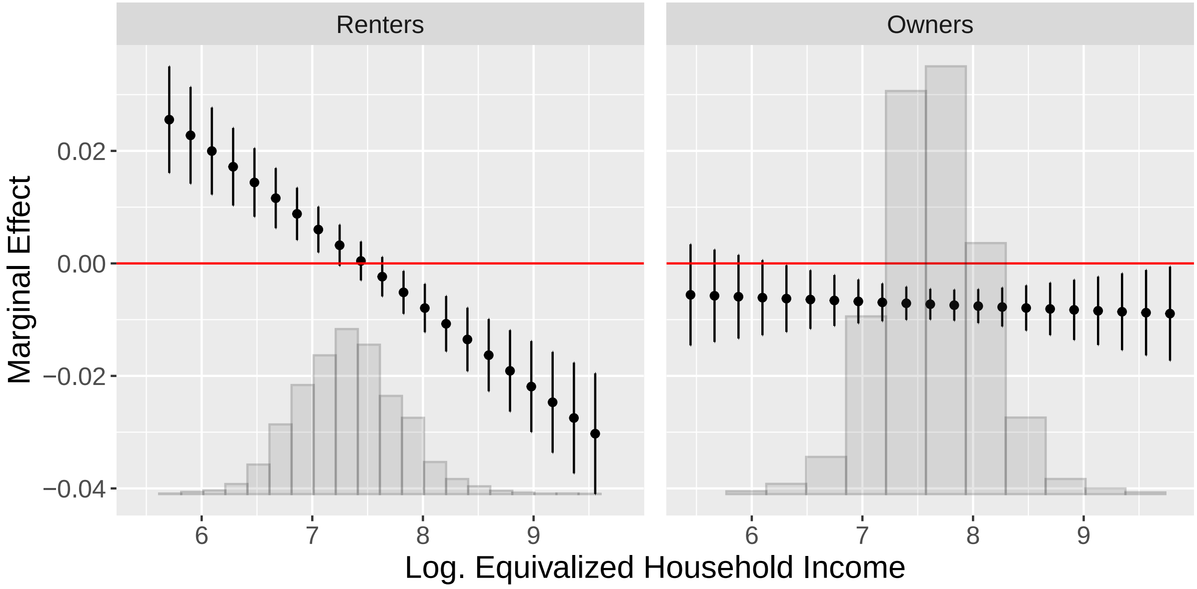

Figure D.17: Conditional marginal effects of local market rents on renters' probability of AfD support as a function of logged equivalized household incomes for medium-to-long-term resident renters (left) and owners (right).

D.5.3. Short-to-medium-term residents: < 5 years at address

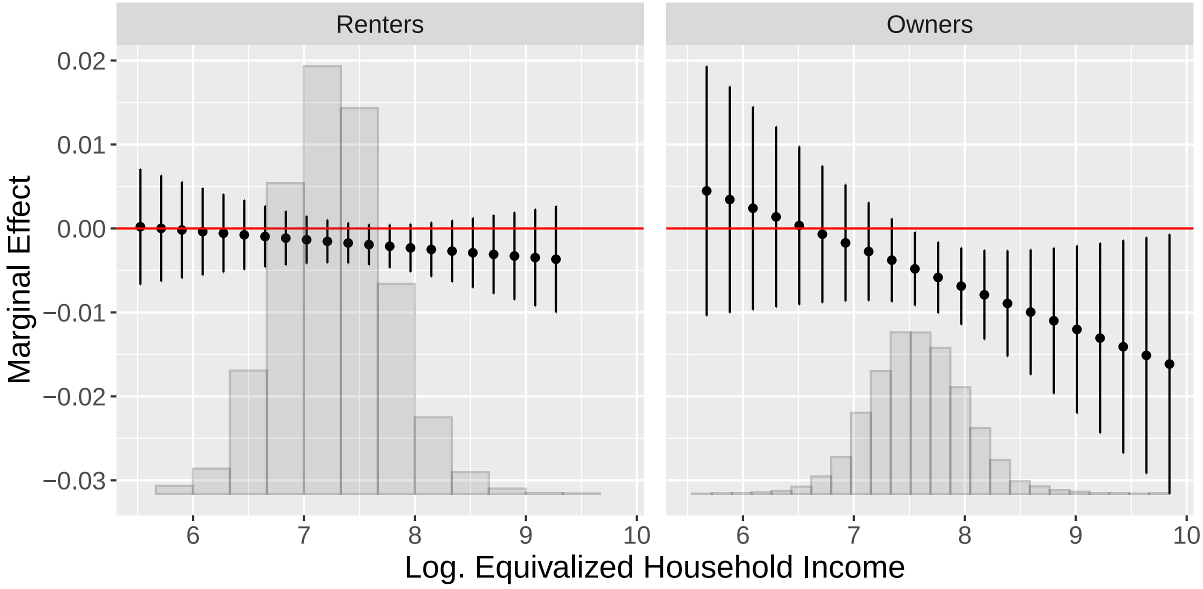

Figure D.18: Conditional marginal effects of local market rents on renters' probability of AfD support as a function of logged equivalized household incomes for short-to-medium-term resident renters (left) and owners (right).

D.5.4. Short-term residents: < 3 years at address

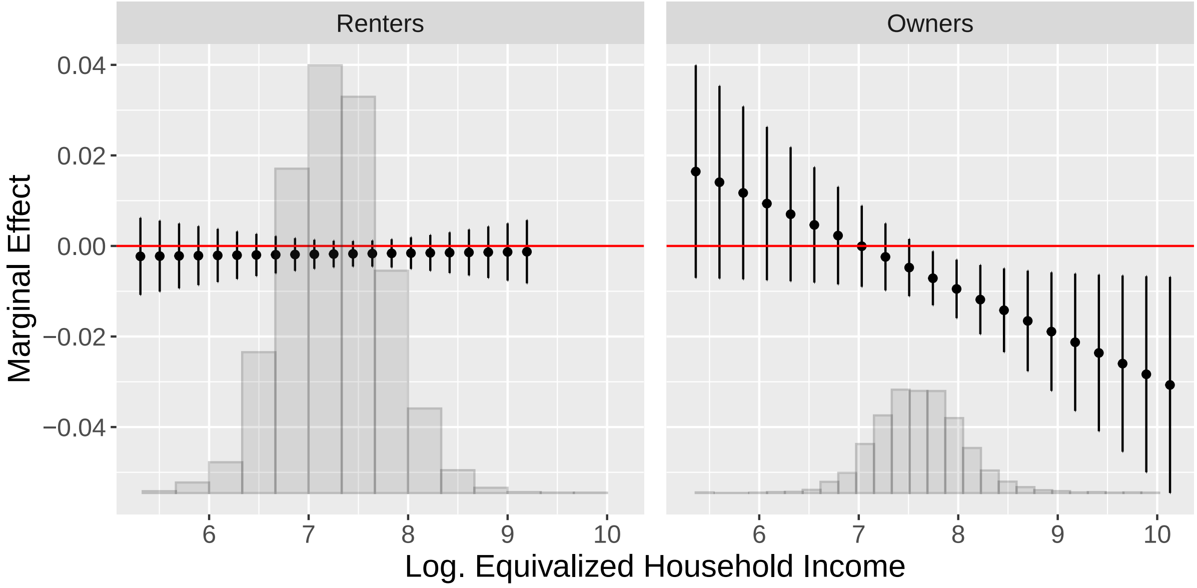

Figure D.19: Conditional marginal effects of local market rents on renters' probability of AfD support as a function of logged equivalized household incomes for short-term resident renters (left) and owners (right).

D.6. Robustness: Fixed-effects model

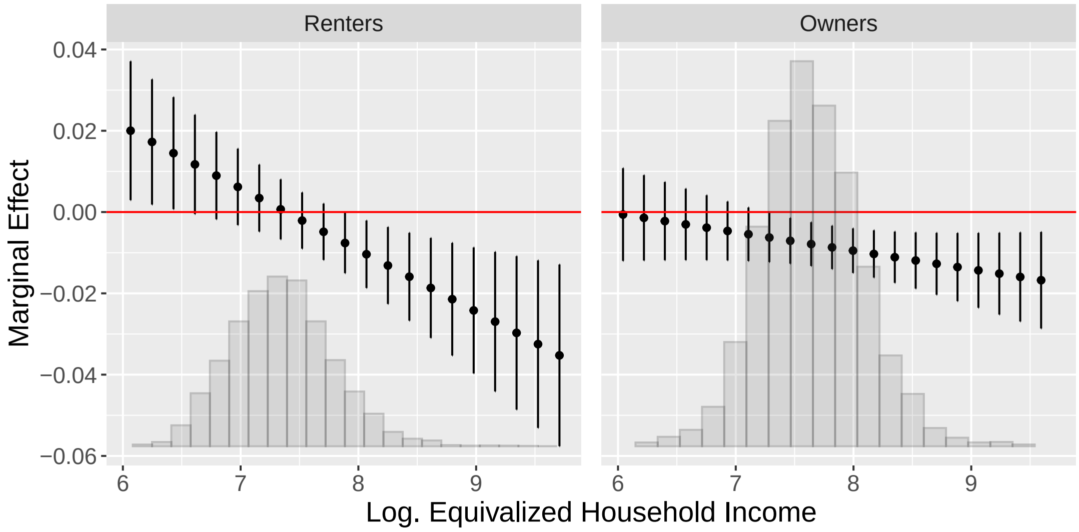

Figure D.20: Conditional marginal effects of local market rents on renters' probability of AfD support as a function of logged equivalized household incomes for long-term resident renters (left) and owners (right). Estimates from a fixed effects model with standard errors clustered at the levels of respondents and postcode areas. The fixed effects model includes all covariates that enter the within-between models in our main analysis as within predictors, as well as respondents' 2014-2018 mean logged equivalized household income as a time-invariant moderator.

D.7. Robustness: Inflation-adjusted measures

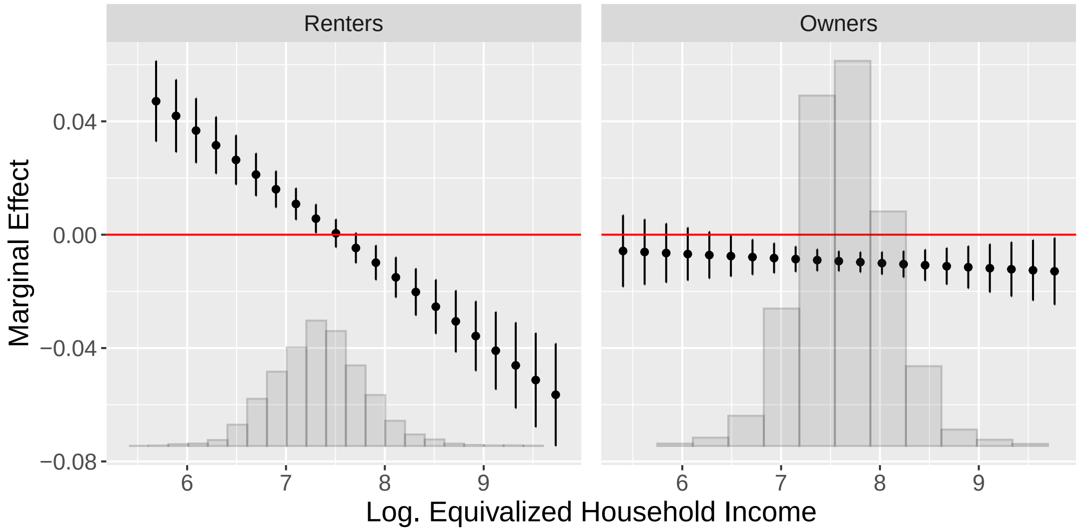

Figure D.21: Conditional marginal effects of local market rents on renters' probability of AfD support as a function of logged equivalized household incomes for long-term resident renters (left) and owners (right). Estimates from a fixed effects model with standard errors clustered at the levels of respondents and postcode areas. Unlike the models in the main specification, this specification uses inflation-adjusted versions of local market rents (per sqm), household rents (per sqm), and household incomes. Adjustment factors are based on official 2014-2018 data on annual consumer price indices provided by the Federal Statistical Office.

E. Descriptive characterization of renter and owner subsets

Below, we show descriptive characterizations of various renter (Table E.1) and homeowner (Table E.2) subsets, based on our analytical 2014-2018 sample from the GSOEP. Each table shows the subgroup-specific means/proportions of the variables listed in the leading column 'Outcomes' with their corresponding 95% confidence intervals. The subsets can be interpreted as follows:

- *All*: All residents
- *Less than 3 years*: Residents who moved to their current address within three years of the interview date
- *Less than 5 years*: Residents who moved to their current address within five years of the interview date
- *Since 3 years*: Residents who moved to their current address at least three years before the interview date
- *Since 5 years*: Residents who moved to their current address at least five years before the interview date

Outcome	All	Less than 3 years	Less than 5 years	Since 3 years	Since 5 years
Women	53.1 [51.6, 54.6]	52.6 [50.4, 54.8]	52.4 [50.6, 54.2]	53.3 [51.6, 55.0]	53.9 [51.9, 55.9]
Age	49.1 [48.5, 49.6]	39.5 [38.8, 40.1]	42.6 [42.0, 43.2]	53.7 [53.1, 54.3]	56.7 [56.0, 57.4]
Household members	2.1 [2.0, 2.1]	2.1 [2.1, 2.2]	2.1 [2.1, 2.2]	2.0 [2.0, 2.1]	1.9 [1.9, 2.0]
Proportion econ. active household members	50.4 [49.3, 51.5]	57.2 [55.7, 58.6]	54.8 [53.6, 56.1]	47.2 [45.8, 48.5]	45.2 [43.6, 46.9]
Log. monthly household income (equiv.)	7.3 [7.3, 7.4]	7.3 [7.3, 7.3]	7.3 [7.3, 7.3]	7.4 [7.3, 7.4]	7.4 [7.3, 7.4]
Education: Less than upper-secondary	13.9 [13.0, 14.9]	15.2 [13.8, 16.7]	15.2 [14.0, 16.4]	13.3 [12.2, 14.4]	12.4 [11.2, 13.7]
Education: Secondary	50.6 [49.2, 52.1]	45.3 [43.2, 47.3]	46.9 [45.2, 48.6]	53.2 [51.6, 54.9]	55.0 [53.0, 56.9]
Education: Post-secondary non-tertiary	7.6 [6.8, 8.4]	9.6 [8.3, 10.8]	9.0 [8.0, 10.0]	6.6 [5.8, 7.5]	6.0 [5.0, 6.9]
Education: Higher vocational	6.1 [5.3, 6.8]	4.4 [3.5, 5.2]	4.9 [4.2, 5.7]	6.9 [6.0, 7.8]	7.4 [6.3, 8.5]
Education: Tertiary	21.8 [20.6, 23.0]	25.6 [23.6, 27.6]	24.0 [22.4, 25.5]	19.9 [18.6, 21.2]	19.2 [17.7, 20.7]
Employment: Atypical	15.7 [14.9, 16.4]	22.4 [21.1, 23.7]	19.8 [18.8, 20.9]	12.4 [11.6, 13.2]	10.8 [9.8, 11.7]
Employment: In Education	3.1 [2.7, 3.4]	5.1 [4.4, 5.8]	4.3 [3.7, 4.8]	2.1 [1.7, 2.5]	1.7 [1.3, 2.1]
Employment: Inactive	8.6 [7.9, 9.2]	7.9 [7.1, 8.7]	8.3 [7.6, 9.0]	8.9 [8.1, 9.7]	8.9 [8.0, 9.8]
Employment: Retired	20.7 [19.5, 21.8]	9.2 [8.2, 10.3]	12.5 [11.5, 13.6]	26.2 [24.8, 27.6]	30.2 [28.5, 31.9]
Employment: Unemployed	6.3 [5.7, 6.8]	7.3 [6.4, 8.1]	6.9 [6.2, 7.6]	5.8 [5.1, 6.4]	5.5 [4.7, 6.2]
Local market rent (EUR/sqm)	7.8 [7.7, 7.9]	7.9 [7.8, 8.0]	7.9 [7.8, 8.0]	7.8 [7.7, 7.9]	7.7 [7.6, 7.8]
Household rent (EUR/sqm)	6.9 [6.8, 7.0]	7.4 [7.2, 7.5]	7.2 [7.1, 7.3]	6.7 [6.6, 6.8]	6.6 [6.5, 6.7]
Locality: Rural	37.7 [36.3, 39.0]	37.7 [35.7, 39.7]	37.3 [35.7, 38.9]	37.7 [36.0, 39.3]	38.1 [36.1, 40.1]
Locality: Suburban	18.3 [17.1, 19.4]	19.3 [17.7, 20.9]	19.3 [17.9, 20.7]	17.8 [16.4, 19.1]	17.1 [15.5, 18.6]
Locality: Urban	44.1 [42.6, 45.5]	43.0 [40.9, 45.1]	43.4 [41.7, 45.2]	44.6 [42.9, 46.3]	44.8 [42.8, 46.8]
County-level non-citizens	11.3 [11.1, 11.5]	11.3 [11.1, 11.6]	11.4 [11.2, 11.6]	11.3 [11.1, 11.5]	11.2 [10.9, 11.4]
County-level unemployment	6.8 [6.8, 6.9]	6.7 [6.6, 6.8]	6.7 [6.6, 6.8]	6.9 [6.8, 7.0]	7.0 [6.8, 7.1]
Lives in East Germany	23.4 [22.2, 24.6]	19.7 [18.1, 21.4]	20.3 [19.0, 21.7]	25.2 [23.8, 26.6]	27.0 [25.3, 28.8]
Supports AfD	2.2 [1.8, 2.5]	1.8 [1.3, 2.3]	1.8 [1.4, 2.2]	2.4 [1.9, 2.8]	2.6 [2.0, 3.1]
Voted for AfD	4.7 [4.2, 5.2]	4.0 [3.2, 4.8]	3.8 [3.2, 4.3]	5.0 [4.4, 5.7]	5.8 [4.9, 6.6]
Supports CDU/CSU	13.5 [12.6, 14.3]	12.7 [11.4, 14.0]	13.0 [12.0, 14.1]	13.8 [12.8, 14.8]	14.0 [12.8, 15.2]
Supports FDP	1.1 [0.9, 1.3]	1.3 [1.0, 1.7]	1.2 [0.9, 1.5]	1.0 [0.7, 1.2]	1.0 [0.7, 1.2]
Supports Greens	6.1 [5.5, 6.7]	7.8 [6.7, 8.9]	7.2 [6.4, 8.1]	5.3 [4.7, 5.9]	4.8 [4.0, 5.6]
Supports The Left	4.6 [4.1, 5.1]	4.0 [3.3, 4.8]	4.0 [3.4, 4.6]	4.9 [4.2, 5.5]	5.3 [4.5, 6.1]
Supports SPD	13.2 [12.4, 14.0]	11.3 [10.1, 12.4]	12.2 [11.2, 13.2]	14.1 [13.1, 15.1]	14.4 [13.2, 15.6]
Supports left-wing parties	23.9 [22.8, 25.0]	23.1 [21.5, 24.7]	23.4 [22.1, 24.7]	24.3 [23.0, 25.6]	24.5 [23.0, 26.1]
Supports right-wing parties	16.7 [15.8, 17.7]	15.8 [14.5, 17.2]	16.1 [14.9, 17.2]	17.2 [16.1, 18.3]	17.5 [16.2, 18.9]
Supports no party	57.4 [56.2, 58.6]	59.1 [57.3, 60.9]	58.5 [57.0, 60.0]	56.6 [55.1, 58.0]	56.1 [54.4, 57.8]
n (full)	45,990	15,178	25,932	30,812	20,058
n (vote choice)	18,564	6,801	11,144	11,763	7,420

Table E.1: Characterization of renters (all) and renter subsets in terms of covariates: Means/proportions and 95% confidence intervals.

Outcome	All	Less than 3 years	Less than 5 years	Since 3 years	Since 5 years
Women	50.1 [48.8, 51.5]	49.9 [47.0, 52.9]	50.0 [48.1, 51.9]	50.2 [48.8, 51.5]	50.2 [48.7, 51.7]
Age	55.8 [55.4, 56.3]	44.9 [44.1, 45.6]	49.9 [49.3, 50.5]	56.9 [56.5, 57.4]	58.0 [57.5, 58.5]
Household members	2.5 [2.5, 2.5]	2.7 [2.7, 2.8]	2.7 [2.6, 2.7]	2.5 [2.5, 2.5]	2.5 [2.4, 2.5]
Proportion econ. active household members	46.9 [46.0, 47.9]	57.0 [55.3, 58.6]	52.1 [50.9, 53.4]	45.9 [44.9, 47.0]	45.1 [43.9, 46.2]
Log. monthly household income (equiv.)	7.6 [7.6, 7.6]	7.7 [7.7, 7.7]	7.6 [7.6, 7.7]	7.6 [7.6, 7.6]	7.6 [7.6, 7.6]
Education: Less than upper-secondary	8.9 [8.2, 9.6]	9.2 [7.7, 10.8]	9.9 [8.9, 10.9]	8.9 [8.1, 9.6]	8.5 [7.7, 9.4]
Education: Secondary	51.0 [49.7, 52.3]	41.3 [38.4, 44.1]	46.7 [44.8, 48.5]	52.0 [50.6, 53.3]	52.5 [51.1, 54.0]
Education: Post-secondary non-tertiary	7.3 [6.6, 8.0]	11.4 [9.5, 13.2]	9.4 [8.2, 10.5]	6.9 [6.2, 7.6]	6.6 [5.8, 7.3]
Education: Higher vocational	8.4 [7.7, 9.1]	8.4 [6.7, 10.0]	7.2 [6.2, 8.1]	8.4 [7.6, 9.1]	8.8 [8.0, 9.7]
Education: Tertiary	24.4 [23.3, 25.5]	29.8 [27.0, 32.5]	26.9 [25.1, 28.6]	23.9 [22.7, 25.0]	23.5 [22.3, 24.7]
Employment: Atypical	8.9 [8.3, 9.5]	13.4 [11.8, 15.0]	11.3 [10.3, 12.2]	8.4 [7.9, 9.0]	8.0 [7.5, 8.6]
Employment: In Education	2.0 [1.7, 2.3]	1.5 [1.1, 1.9]	1.8 [1.5, 2.2]	2.1 [1.8, 2.4]	2.1 [1.7, 2.4]
Employment: Inactive	8.6 [8.1, 9.2]	7.0 [6.0, 8.0]	7.8 [7.1, 8.5]	8.8 [8.2, 9.4]	8.9 [8.3, 9.6]
Employment: Retired	28.6 [27.4, 29.7]	10.6 [9.3, 11.9]	18.8 [17.5, 20.1]	30.4 [29.1, 31.6]	32.1 [30.7, 33.4]
Employment: Unemployed	1.2 [1.0, 1.4]	1.5 [1.1, 1.9]	1.3 [1.1, 1.6]	1.2 [1.0, 1.4]	1.2 [1.0, 1.4]
Local market rent (EUR/sqm)	7.3 [7.2, 7.3]	7.5 [7.3, 7.6]	7.3 [7.3, 7.4]	7.3 [7.2, 7.3]	7.2 [7.2, 7.3]
Locality: Rural	51.1 [49.8, 52.4]	49.1 [46.2, 52.0]	49.0 [47.1, 50.9]	51.3 [49.9, 52.6]	51.8 [50.3, 53.3]
Locality: Suburban	26.9 [25.7, 28.1]	27.1 [24.5, 29.7]	27.5 [25.8, 29.1]	26.9 [25.6, 28.1]	26.7 [25.3, 28.0]
Locality: Urban	22.1 [20.9, 23.2]	23.8 [21.4, 26.2]	23.5 [21.8, 25.2]	21.9 [20.7, 23.1]	21.5 [20.3, 22.8]
County-level non-citizens	10.1 [10.0, 10.2]	10.5 [10.2, 10.8]	10.3 [10.1, 10.5]	10.0 [9.9, 10.2]	10.0 [9.9, 10.2]
County-level unemployment	5.8 [5.7, 5.8]	5.8 [5.6, 5.9]	5.8 [5.7, 5.9]	5.8 [5.7, 5.8]	5.8 [5.7, 5.8]
Lives in East Germany	15.0 [14.1, 15.9]	11.5 [9.7, 13.2]	12.4 [11.3, 13.6]	15.3 [14.4, 16.3]	15.9 [14.9, 16.9]
Supports AfD	1.8 [1.6, 2.1]	1.7 [1.0, 2.4]	1.5 [1.1, 1.8]	1.8 [1.6, 2.1]	1.9 [1.6, 2.2]
Voted for AfD	4.8 [4.3, 5.2]	5.1 [3.6, 6.6]	4.1 [3.3, 4.9]	4.7 [4.2, 5.2]	5.0 [4.5, 5.6]
Supports CDU/CSU	23.1 [22.1, 24.0]	20.1 [17.9, 22.3]	22.0 [20.7, 23.4]	23.4 [22.4, 24.4]	23.5 [22.3, 24.6]
Supports FDP	2.0 [1.8, 2.3]	1.9 [1.1, 2.8]	2.0 [1.5, 2.5]	2.1 [1.8, 2.3]	2.1 [1.8, 2.4]
Supports Greens	6.7 [6.1, 7.3]	7.6 [6.2, 9.1]	7.5 [6.6, 8.4]	6.6 [6.0, 7.2]	6.4 [5.8, 7.1]
Supports The Left	2.1 [1.8, 2.4]	2.3 [1.7, 3.0]	2.3 [1.8, 2.7]	2.1 [1.8, 2.4]	2.0 [1.7, 2.4]
Supports SPD	14.2 [13.4, 15.0]	13.5 [11.7, 15.2]	14.5 [13.3, 15.7]	14.3 [13.5, 15.1]	14.1 [13.2, 15.0]
Supports left-wing parties	23.0 [22.1, 24.0]	23.4 [21.2, 25.6]	24.3 [22.8, 25.7]	23.0 [22.0, 24.0]	22.6 [21.5, 23.7]
Supports right-wing parties	26.9 [25.9, 27.9]	23.8 [21.4, 26.1]	25.5 [24.0, 26.9]	27.3 [26.2, 28.3]	27.5 [26.3, 28.6]
Supports no party	48.3 [47.2, 49.4]	50.3 [47.7, 53.0]	48.6 [47.0, 50.3]	48.1 [47.0, 49.2]	48.2 [47.0, 49.5]
n (full)	53,396	5,800	15,835	47,596	37,561
n (vote choice)	21,348	2,981	7,467	18,367	13,881

Table E.2: Characterization of owners (all) and owner subsets in terms of covariates: Means/proportions and 95% confidence intervals.

F. Regression models

F.1. Details

We estimate variants of linear within-between models (Bell and Jones, 2015) at the individual-level with additional random effects to account for spatial dependencies at the postcode and city/county levels. We implement within-between decompositions for most time-varying predictors. Coefficients for the within-demeaned predictors indicate *within effects* whereas the coefficients for the respondent means indicate *between effects* in Tables F.3 and F.4 below. Additionally, for time-invariant variables (like gender and, given that we focus on long-term residents, locality type) as well as so-called 'sluggish' variables that hardly change over time (like educational degrees), we include the original (undemeaned) versions, whose coefficients we denote as *mixed effects*. These also apply to our year fixed effects.

Multiplicative interaction effects of local market rents and household incomes (as well as analogous interactions of actual household rents and household incomes) are products of the non-demeaned levels of household incomes and within-demeaned rent variables (conditional within effects) or respondent-means of the rent variables (conditional between effects), respectively. This specification allows us to assess how the within and between effects of rents vary as a function of the time-varying levels of equivalized household incomes.

F.2. Tables

Variables	Renter model	Owner model
Within effects		
Local market rent (EUR/sqm)	0.160<br>[0.111, 0.208]	0.002<br>[−0.034, 0.038]
Equiv. household income (log)	−0.006<br>[−0.022, 0.010]	0.002<br>[−0.007, 0.012]
Proportion personal income	−0.023<br>[−0.043, −0.003]	−0.020<br>[−0.036, −0.006]
Proportion econ. active household members	−0.043<br>[−0.062, −0.024]	0.004<br>[−0.009, 0.016]
Number of household members	−0.019<br>[−0.027, −0.010]	−0.009<br>[−0.014, −0.003]
Labor market status (ref: Full-time employment)		
Atypical employment	−0.003<br>[−0.012, 0.006]	−0.004<br>[−0.013, 0.004]
Economically inactive	−0.030<br>[−0.047, −0.013]	0.001<br>[−0.009, 0.011]
Unemployed	−0.032<br>[−0.051, −0.014]	−0.013<br>[−0.029, 0.003]
In education	−0.019<br>[−0.044, 0.007]	−0.018<br>[−0.035, −0.002]
Retired	−0.043<br>[−0.063, −0.023]	−0.006<br>[−0.019, 0.007]
Household rent (EUR/sqm)	0.010<br>[−0.017, 0.038]	
Local market rent (demeaned) × household income	−0.021<br>[−0.028, −0.015]	−0.001<br>[−0.006, 0.003]
Household rent (demeaned) × household income	−0.002<br>[−0.005, 0.002]	
Mixed effects		
Intercept	0.043<br>[−0.158, 0.244]	0.050<br>[−0.069, 0.175]
Age	0.000<br>[0.000, 0.000]	0.000<br>[0.000, 0.000]
East/West residence (ref: West)		
East	0.010<br>[0.000, 0.019]	0.021<br>[0.014, 0.029]
Sex (ref: Male)		
Female	−0.021<br>[−0.028, −0.014]	−0.018<br>[−0.023, −0.013]

Education (ref: < Upper secondary)		
Upper-secondary	0.007	−0.006
	[−0.004, 0.018]	[−0.016, 0.003]
Post-secondary non-tertiary	−0.004	−0.007
	[−0.019, 0.012]	[−0.021, 0.005]
Higher vocational	0.000	−0.014
	[−0.019, 0.021]	[−0.025, −0.004]
Tertiary	−0.009	−0.012
	[−0.023, 0.004]	[−0.024, −0.002]
Locality (ref: Rural)		
Suburban	0.009	0.009
	[−0.003, 0.020]	[0.002, 0.016]
Urban	−0.004	0.007
	[−0.014, 0.005]	[−0.001, 0.015]
Year (ref: 2014)		
2015	0.002	0.008
	[−0.004, 0.008]	[0.004, 0.012]
2016	0.013	0.018
	[0.006, 0.019]	[0.014, 0.023]
2017	0.012	0.022
	[0.004, 0.020]	[0.017, 0.027]
2018	0.017	0.029
	[0.008, 0.026]	[0.023, 0.036]
Between effects		
Equiv. household income (log)	−0.003	−0.004
	[−0.029, 0.024]	[−0.020, 0.011]
Local market rent (EUR/sqm)	−0.005	−0.001
	[−0.026, 0.016]	[−0.016, 0.014]
Proportion personal income	0.002	−0.002
	[−0.013, 0.016]	[−0.011, 0.007]
Proportion econ. active household members	−0.003	0.000
	[−0.025, 0.018]	[−0.013, 0.013]
Number of household members	−0.001	0.000
	[−0.006, 0.003]	[−0.003, 0.003]
Labor market status (ref: Full-time employment)		
Atypical employment	0.003	−0.004
	[−0.012, 0.018]	[−0.015, 0.007]
Economically inactive	0.000	0.004
	[−0.020, 0.020]	[−0.008, 0.016]
Unemployed	0.000	0.001
	[−0.024, 0.024]	[−0.029, 0.029]

In education	0.005	-0.013
	$[-0.028, 0.038]$	$[-0.034, 0.007]$
Retired	-0.015	-0.011
	$[-0.036, 0.006]$	$[-0.023, 0.001]$
Household rent (EUR/sqm)	-0.001	
	$[-0.026, 0.024]$	
Local market rent (mean) × household income	0.001	0.000
	$[-0.002, 0.004]$	$[-0.002, 0.002]$
Household rent (mean) × household income	0.000	
	$[-0.003, 0.003]$	
N		
Observations	20058	37561
Individuals	7411	11442
Postcode areas	2341	2708
Counties/cities	390	397
Standard deviations of intercepts		
$\sigma_{\text{Individuals}}$	0.092	0.075
$\sigma_{\text{Counties/cities}}$	0.010	0.002
$\sigma_{\text{Postcode areas}}$	0.033	0.037
$\sigma_{\text{Observations}}$	0.116	0.108

Table F.3: Coefficients and simulation-based 95% confidence intervals from hierarchical linear within-between models, estimated across $M = 5$ imputations.

Variables	Renter model	Owner model
Within effects		
Local market rent (EUR/sqm)	0.160<br>[0.111, 0.208]	0.002<br>[−0.034, 0.038]
Equiv. household income (log)	−0.006<br>[−0.022, 0.010]	0.002<br>[−0.007, 0.012]
Proportion personal income	−0.023<br>[−0.043, −0.003]	−0.020<br>[−0.036, −0.006]
Proportion econ. active household members	−0.043<br>[−0.062, −0.024]	0.004<br>[−0.009, 0.016]
Number of household members	−0.019<br>[−0.027, −0.010]	−0.009<br>[−0.014, −0.003]
Labor market status (ref: Full-time employment)		
Atypical employment	−0.003<br>[−0.012, 0.006]	−0.004<br>[−0.013, 0.004]
Economically inactive	−0.030<br>[−0.047, −0.013]	0.001<br>[−0.009, 0.011]
Unemployed	−0.032<br>[−0.051, −0.014]	−0.013<br>[−0.029, 0.003]
In education	−0.019<br>[−0.044, 0.007]	−0.018<br>[−0.035, −0.002]
Retired	−0.043<br>[−0.063, −0.023]	−0.006<br>[−0.019, 0.007]
Household rent (EUR/sqm)	0.010<br>[−0.017, 0.038]	
Local market rent (demeaned) × household income	−0.021<br>[−0.028, −0.015]	−0.001<br>[−0.006, 0.003]
Household rent (demeaned) × household income	−0.002<br>[−0.005, 0.002]	
Mixed effects		
Intercept	0.043<br>[−0.158, 0.244]	0.050<br>[−0.069, 0.175]
Age	0.000<br>[0.000, 0.000]	0.000<br>[0.000, 0.000]
East/West residence (ref: West)		
East	0.010<br>[0.000, 0.019]	0.021<br>[0.014, 0.029]
Sex (ref: Male)		
Female	−0.021<br>[−0.028, −0.014]	−0.018<br>[−0.023, −0.013]
Education (ref: < Upper secondary)		

Upper-secondary	0.007<br>[−0.004, 0.018]	−0.006<br>[−0.016, 0.003]
Post-secondary non-tertiary	−0.004<br>[−0.019, 0.012]	−0.007<br>[−0.021, 0.005]
Higher vocational	0.000<br>[−0.019, 0.021]	−0.014<br>[−0.025, −0.004]
Tertiary	−0.009<br>[−0.023, 0.004]	−0.012<br>[−0.024, −0.002]
Locality (ref: Rural)		
Suburban	0.009<br>[−0.003, 0.020]	0.009<br>[0.002, 0.016]
Urban	−0.004<br>[−0.014, 0.005]	0.007<br>[−0.001, 0.015]
Year (ref: 2014)		
2015	0.002<br>[−0.004, 0.008]	0.008<br>[0.004, 0.012]
2016	0.013<br>[0.006, 0.019]	0.018<br>[0.014, 0.023]
2017	0.012<br>[0.004, 0.020]	0.022<br>[0.017, 0.027]
2018	0.017<br>[0.008, 0.026]	0.029<br>[0.023, 0.036]
Between effects		
Equiv. household income (log)	−0.003<br>[−0.029, 0.024]	−0.004<br>[−0.020, 0.011]
Local market rent (EUR/sqm)	−0.005<br>[−0.026, 0.016]	−0.001<br>[−0.016, 0.014]
Proportion personal income	0.002<br>[−0.013, 0.016]	−0.002<br>[−0.011, 0.007]
Proportion econ. active household members	−0.003<br>[−0.025, 0.018]	0.000<br>[−0.013, 0.013]
Number of household members	−0.001<br>[−0.006, 0.003]	0.000<br>[−0.003, 0.003]
Labor market status (ref: Full-time employment)		
Atypical employment	0.003<br>[−0.012, 0.018]	−0.004<br>[−0.015, 0.007]
Economically inactive	0.000<br>[−0.020, 0.020]	0.004<br>[−0.008, 0.016]
Unemployed	0.000<br>[−0.024, 0.024]	0.001<br>[−0.029, 0.029]

In education	0.005	-0.013
	$[-0.028, 0.038]$	$[-0.034, 0.007]$
Retired	-0.015	-0.011
	$[-0.036, 0.006]$	$[-0.023, 0.001]$
Household rent (EUR/sqm)	-0.001	
	$[-0.026, 0.024]$	
Local market rent (mean) $\times$ household income	0.001	0.000
	$[-0.002, 0.004]$	$[-0.002, 0.002]$
Household rent (mean) $\times$ household income	0.000	
	$[-0.003, 0.003]$	
N		
Observations	20058	37561
Individuals	7411	11442
Postcode areas	2341	2708
Counties/cities	390	397
Standard deviations of intercepts		
$\sigma_{\text{Individuals}}$	0.092	0.075
$\sigma_{\text{Counties/cities}}$	0.010	0.002
$\sigma_{\text{Postcode areas}}$	0.033	0.037
$\sigma_{\text{Observations}}$	0.116	0.108

Table F.4: Coefficients and simulation-based 95% confidence intervals from hierarchical linear within-between models, estimated across $M = 5$ imputations.

References

Abou-Chadi, Tarik, Silja Häusermann, Reto Mitteregger, Nadja Mosimann and Markus Wagner. 2024. "Trade-offs of social democratic party strategies in a pluralized issue space: a conjoint analysis." *World Politics* .

Baldenius, Till, Sebastian Kohl and Moritz Schularick. 2020. "Die neue Wohnungsfrage - Gewinner und Verlierer des deutschen Immobilienbooms." *Leviathan* 48(2):195–236.

Bell, Andrew and Kelvyn Jones. 2015. "Explaining Fixed Effects: Random Effects Modeling of Time-Series Cross-Sectional and Panel Data." *Political Science Research and Methods* 3(1):133–151.

Imai, Kosuke, Luke Keele and Dustin Tingley. 2010. "A general approach to causal mediation analysis." *Psychological methods* 15(4):309–34.
URL: *http://www.ncbi.nlm.nih.gov/pubmed/20954780*

Imai, Kosuke, Luke Keele, Dustin Tingley and Teppei Yamamoto. 2010. "Causal mediation analysis using R." *Advances in social science research using R* pp. 129–154.

RWI; ImmobilienScout24. 2021*a*. "RWI Real Estate Data - Apartments for Rent - SUF. RWI-GEO-RED. Version: 1.".
URL: *https://doi.org/10.7807/immo:red:wm:suf:v5*

RWI; ImmobilienScout24. 2021*b*. "RWI Real Estate Data - Houses for Rent - SUF. RWI-GEO-RED. Version: 1.".
URL: *https://doi.org/10.7807/immo:red:hm:suf:v5*
